# ConsAlign: simultaneous RNA structural aligner based on rich transfer learning and thermodynamic ensemble model of alignment scoring

**DOI:** 10.1093/bioinformatics/btad255

**Published:** 2023-04-19

**Authors:** Masaki Tagashira

**Affiliations:** Department of Computational Biology and Medical Sciences, University of Tokyo, Chiba 277-8561, Japan

## Abstract

**Motivation:**

To capture structural homology in RNAs, alignment and folding (AF) of RNA homologs has been a fundamental framework around RNA science. Learning sufficient scoring parameters for simultaneous AF (SAF) is an undeveloped subject because evaluating them is computationally expensive.

**Results:**

We developed ConsTrain—a gradient-based machine learning method for rich SAF scoring. We also implemented ConsAlign—a SAF tool composed of ConsTrain’s learned scoring parameters. To aim for better AF quality, ConsAlign employs (1) transfer learning from well-defined scoring models and (2) the ensemble model between the ConsTrain model and a well-established thermodynamic scoring model. Keeping comparable running time, ConsAlign demonstrated competitive AF prediction quality among current AF tools.

**Availability and implementation:**

Our code and our data are freely available at https://github.com/heartsh/consalign and https://github.com/heartsh/consprob-trained.

## 1 Introduction

Machine learning is applied to each significant bioinformatics subject, including structural bioinformatics. Also, machine learning has attracted computational scientists’ attention around folding single RNA sequences. In folding single RNA sequences, modern parameter training methods optimize secondary structure scoring parameters based on both training RNA sequences and training RNA secondary structures (Do et al. 2006a; Zakov et al. 2011; Rivas et al. 2012; Singh et al. 2019; Sato et al. 2021). ‘Simultaneous alignment and folding’ (SAF) of RNA homolog sequences (Sankoff 1985) is an advanced solution to folding single RNA sequences. SAF improves the folding quality of an RNA homolog sequence, incorporating other RNA homologs concerned. Modern SAF tools include LocARNA (Will et al. 2007), RAF (Do et al. 2008), DAFS (Sato et al. 2012), and SPARSE (Will et al. 2015). Modern SAF tools emphasize lightening their computation to sparsify potential SAF. The machine-learning methods of SAF have fallen behind those of folding single RNA sequences due to the complexity of the former (Dowell and Eddy 2006; Do et al. 2008).

**Figure 1.**
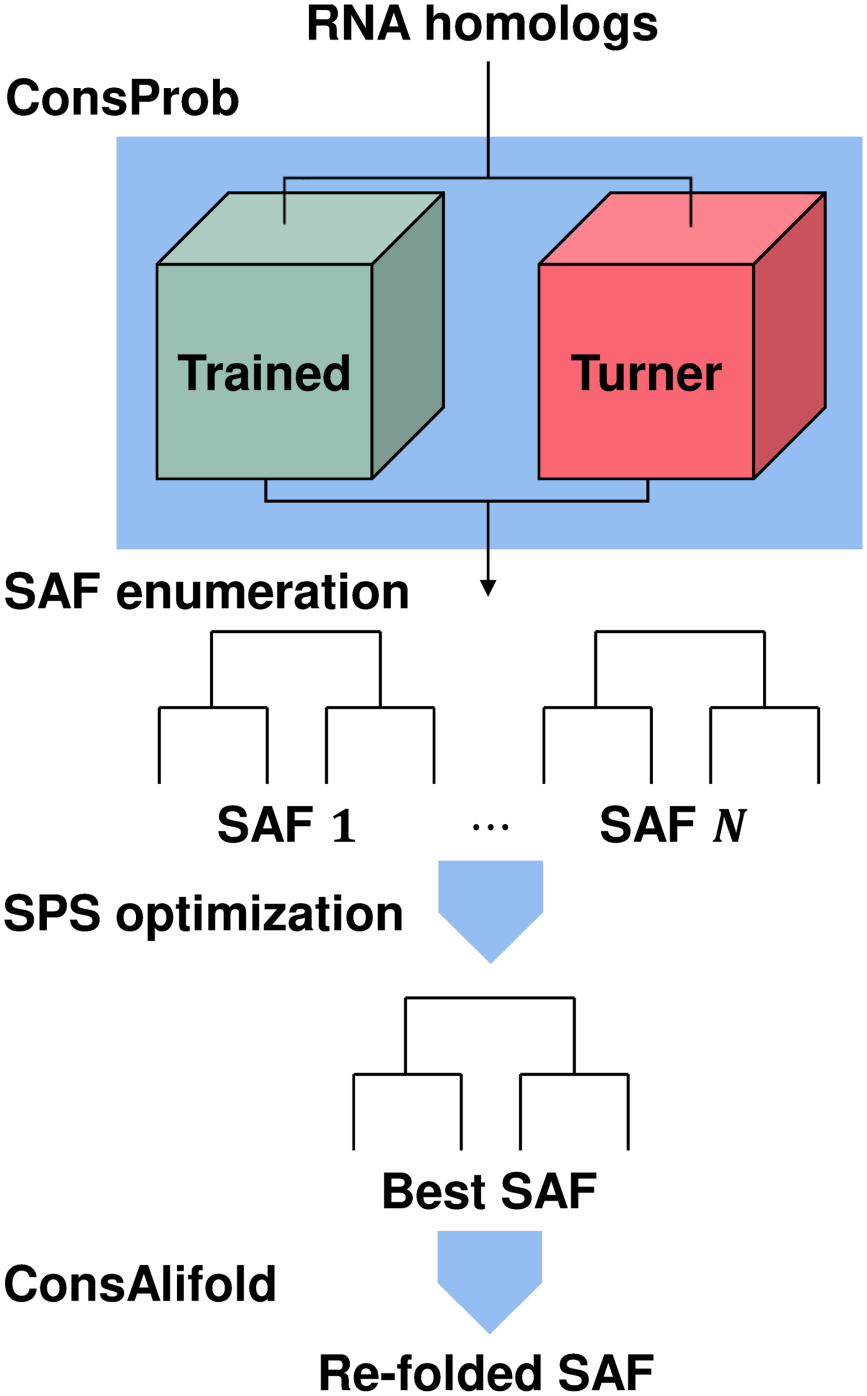
ConsAlign’s work process taking unaligned, unfolded RNA homologs as an input. ConsAlign begins its AF prediction by conducting ConsProb (Tagashira and Asai 2022). ConsProb applies its probability inference based on sparse pairwise SAF to each RNA homolog pair. ConsAlign realizes its robust AF prediction utilizing ConsProb’s two different implementations—ConsProb-trained and ConsProb-Turner. ConsProb-Turner is ConsProb’s physics-based implementation proposed in Tagashira and Asai (2022). ConsProb-trained is ConsProb’s machine learning-based implementation proposed in this study. ConsAlign moves from ConsProb to a candidate SAF enumerating step. ConsAlign predicts the best multiple SAF given each candidate pair of SAF hyper-parameters. From these SAF candidates, the optimal SAF is found by maximizing its expected SPS (Thompson et al. 1999). This optimal SAF is reassessed by trying to reach a more sophisticated consensus of its secondary structures via ConsAlifold (Tagashira and Asai 2022). More specifically, ConsAlifold evaluates all the aligned homologs by utilizing ConsProb and RNAalifold (Bernhart et al. 2008) as different probability inference sources. Finally, ConsAlifold refolds the optimal SAF retaining immature secondary structures to obtain a firm consensus structure.

This study proposes ‘ConsTrain’—an ‘SAF score learning extension’ of our sparse inside-outside algorithm (Tagashira and Asai 2022). In Tagashira and Asai (2022), our sparse inside-outside algorithm scores possible SAF by (the *ad hoc* combination of) the scoring parameters of (i) Turner’s (nearest neighbor physics) model for RNA secondary structures (Turner and Mathews 2010) and (ii) the CONTRAlign model for RNA sequence alignments (Do et al. 2006b). By ConsTrain, we intend to improve the predictive performance of our sparse inside–outside algorithm by seeking a better SAF scoring model than this ad hoc combination. ConsTrain is based on the ‘pairwise conditional log-linear model’ (pair-CLLM)—a flexible discriminative training framework (Do et al. 2006a). Scoring parameter training for SAF has been proposed using not the pair-CLLM but the pairwise stochastic context-free grammar (the pair-SCFG)—a classical generative training framework (Dowell and Eddy 2006). Dowell and Eddy (2006)’s pair-SCFG consists of its simple rules and therefore lacks rules corresponding to significant features in RNA secondary structures (e.g. base pair stacking). In this study, we realize a pair-CLLM—a generalized variant of a pair-SCFG (Do et al. 2006a)—able to capture these significant features. To test the prediction performance of ConsTrain’s learned SAF parameters, we build ‘ConsAlign’—a SAF tool parameterized with them (Fig. 1). ConsAlign conducts sparse dynamic programming (Will et al. 2007; Do et al. 2008; Will et al. 2015) according to the ‘progressive alignment scheme’ (Feng and Doolittle 1987). The progressive alignment scheme aligns and merges intermediate matching profiles along an alignment guide tree. Sparse dynamic programming enables to infer SAF within reasonable computational costs, shrinking the space of potential SAF. ConsAlign depends on its hyper-parameters to control SAF quality but corrects them automatically by our original scheme without exploiting any validation data. As our first essential contribution, we introduce ‘transfer learning’ (Singh et al. 2019) based on conventional trained models into ConsTrain. In practice, the performance of machine learning methods depends on their initialization of trained parameters. We transfer (or copy) the parameter values of conventional trained models into those of our trained scoring model to fully bring out its potential. As our second essential contribution, we also introduce the ‘model ensemble’ (Aghaeepour and Hoos 2013; Singh et al. 2019) between our thermodynamic probability inference and ConsTrain’s learned probability inference into ConsAlign. Each of these probability inference methods has good and bad points in predicting SAF. In predicting SAF, we combine our probability inference methods to take advantage of the strengths that each provides. We expect that ConsAlign will acquire higher precision and more robustness through our essential contributions.

## 2 Methods

We initiate our computational methodology by introducing ConsAlign—our proposed SAF tool. Then, we turn to ConsTrain—our proposed training method for ConsAlign’s SAF parameters. ConsAlign and ConsTrain share ConsProb—our probability inference method based on sparse pairwise SAF (Tagashira and Asai 2022)—as their core sub-routine algorithm.

### 2.1 Our proposed alignment and folding tool to maximize accuracy

#### 2.1.1 Quadratic pairwise SAF guaranteeing its optimality

**Figure 2.**
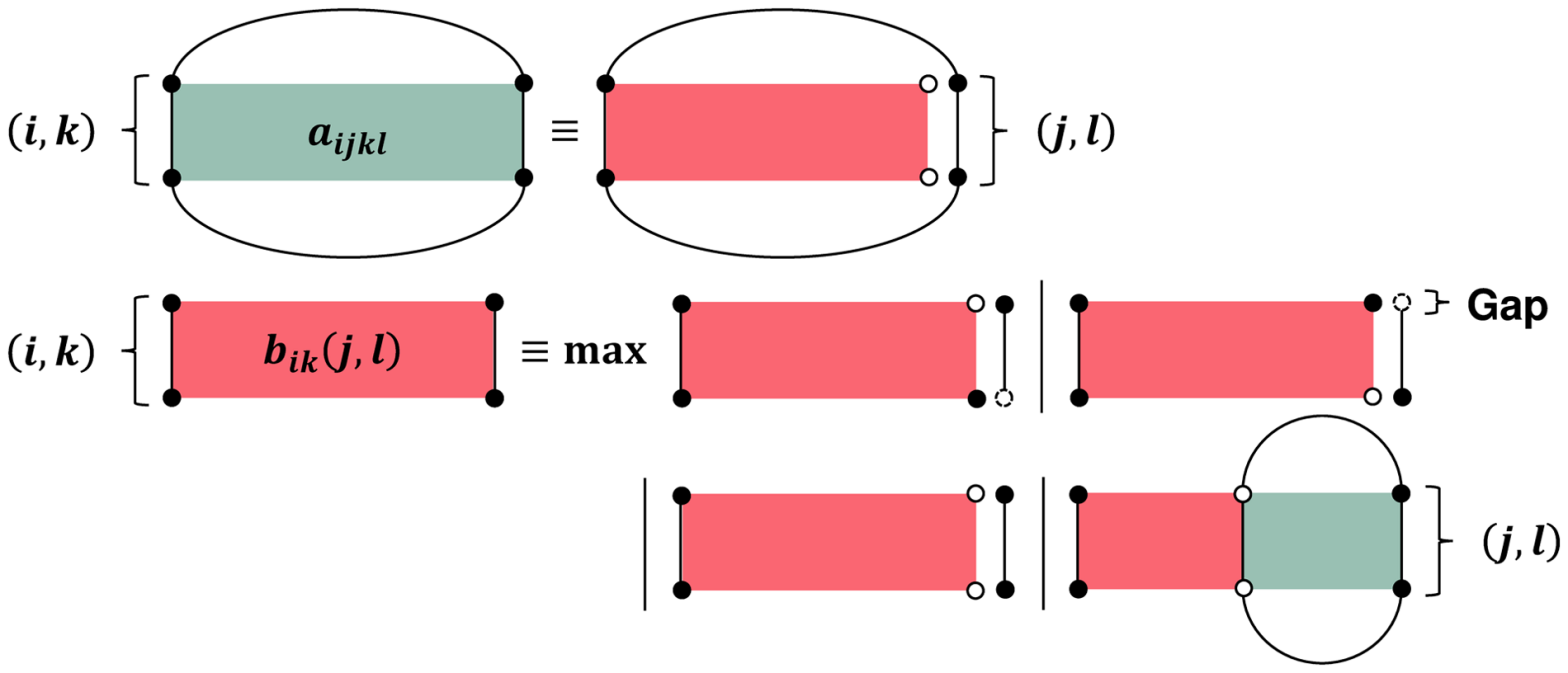
Recursive diagram of our pairwise SAF. *a_ijkl_* represents the best sub-SAF inside the matching between every two base-pairing nucleotide pairs (i,j),(k,l), including (i,j),(k,l). Each dynamic programming cell *a_ijkl_* depends on every dynamic programming cell $b_{ik}(j,l)$, and $b_{ik}(j,l)$ expresses the best sub-SAF from every two fixed nucleotides (*i*, *k*) to every two free nucleotides (*j*, *l*). Each dynamic programming cell $b_{ik}(j,l)$ contains four different cases: (i) each unpaired nucleotide *j* inserted between every two nucleotides l,l+1, (ii) each unpaired nucleotide *l* deleted between every two nucleotides j,j+1, (iii) the matching of every two unpaired nucleotides (*j*, *l*), and (4) the matching of every two paired nucleotides (*j*, *l*). Dynamic programming cells $a_{\mathrm{ijkl}},b_{ik}(j,l)$ call each other, shortening the length of the best sub-SAF.

In the *γ*-centroid estimator principle (Ding et al. 2005; Hamada et al. 2009a, 2009b, 2009c, 2011), we define a *γ*-centroid pairwise SAF recursion like LocARNA (Will et al. 2007) and RAF (Do et al. 2008), as in Fig. 2: starting from the initial condition $b_{ik}(i,k)\leftarrow 0$. We describe the computation of sparse posterior probabilities $p_{ik}^{M}(\theta ),p_{ij}^{P}(\theta )$ in Appendix 1. ConsProb implements our sparse inside–outside algorithm for pairwise SAF and offers different posterior probabilities [e.g. SAF-based variants of structural context profiles computed by CapR (Fukunaga et al. 2014)] based on sparse pairwise SAF, including probabilities $p_{ik}^{M}(\theta ),p_{ij}^{P}(\theta )$ (Tagashira and Asai 2022). $\gamma^{M},\gamma^{P}$ are any two real numbers ˃1. Hyper-parameters $\gamma^{M}$ and $\gamma^{P}$ control the predictive significance of nucleotide matching and nucleotide base-pairing, respectively. We note that ConsProb considers loop energy scores or machine-learned loop scores, whereas ConsAlign depends only on ConsProb’s probabilities derived from these loop-specific scores.


(1) $$\begin{array}{c} a_{\mathrm{ijkl}}\equiv b_{ik}(j-1,l-1)+\alpha_{\mathrm{ijkl}}\\ b_{ik}(j,l)\equiv \max\{\begin{array}{c} b_{ik}(j-1,l)\\ b_{ik}(j,l-1)\\ b_{ik}(j-1,l-1)+\gamma^{M}p_{jl}^{M}(\theta )-1\\ \max_{uv:i<u<j,k<v<l}[b_{ik}(u-1,v-1)+a_{\mathrm{ujvl}}] \end{array}\\ \alpha_{\mathrm{ijkl}}\overset{\text{def}}{=}\gamma^{M}[p_{ik}^{M}(\theta )+p_{jl}^{M}(\theta )]+\gamma^{P}[p_{ij}^{P}(\theta )+p_{kl}^{P}(\theta )]-4 \end{array}$$

We need to conduct Equation (1) with $O(N^{3}M^{3})$ running time and $O(N^{2}M^{2})$ memory usage in naive computation. (*N*, *M* are the lengths of every two RNA sequences.) However, we can perform Equation (1) only with both $O(L^{2})$ running time and *O*(*L*) memory usage if hyper-parameters $\gamma^{M},\gamma^{P}$ are sufficiently small. [*L* is $L\overset{\text{def}}{=}\max(N,M)$.] We can realize this computational complexity reduction since Equation (1) accepts only nucleotide matches (*i*, *k*) satisfying the condition and nucleotide base-pairings (i,j),(k,l) satisfying the conditions and maximizes a form of expected accuracy


(2) $$p_{ik}^{M}(\theta )\geq \frac{1}{\gamma^{M}}\Leftrightarrow \gamma^{M}p_{ik}^{M}(\theta )-1\geq 0$$

(3) $$\begin{array}{cc} & p_{ij}^{P}(\theta )\geq \frac{1}{\gamma^{P}}\Leftrightarrow \gamma^{P}p_{ij}^{P}(\theta )-1\geq 0\\ & p_{kl}^{P}(\theta )\geq \frac{1}{\gamma^{P}}\Leftrightarrow \gamma^{P}p_{kl}^{P}(\theta )-1\geq 0 \end{array}$$

$$\sum_{ik:(i,k)\in M}[\gamma^{M}p_{ik}^{M}(\theta )-1]+\sum_{\mathrm{ijkl}:(i,j,k,l)\in M^{P}}\{\gamma^{P}[p_{ij}^{P}(\theta )+p_{kl}^{P}(\theta )]-2\}.$$

Here, *M* is a set of all matched nucleotide pairs in SAF under optimization, and $M^{P}$ is a set of all matched base-pairing nucleotide quadruples in SAF under optimization. LocARNA and RAF impose Equations (2) and (3) upon possible pairwise SAF to enable both $O(L^{2})$ running time and *O*(*L*) memory usage (Do et al. 2008). However, LocARNA and RAF do not assure that their obtained SAF is optimum (i.e. retaining the best score of all possible SAF candidates) because constraints upon Equations (2) and (3) are not derived from optimized SAF scores naturally. In contrast, Equation (1) naturally leads to constraining SAF under Equations (2) and (3) and assures that obtained SAF is optimum even if we apply these constraints.

#### 2.1.2 Extending pairwise SAF into multiple SAF

Our multiple SAF is based on the progressive SAF scheme (Feng and Doolittle 1987) like conventional SAF tools (Will et al. 2007; Do et al. 2008; Sato et al. 2012; Will et al. 2015; Li et al. 2021). When we build a ‘UPGMA guide tree’ (Sneath and Sokal 1962) from RNA homolog sequences to be aligned, we measure the easy-to-compute similarity between every two RNA homologs


$$\sigma (\theta )\overset{\text{def}}{=}\sum_{ik}p_{ik}^{M}(\theta )I[p_{ik}^{M}(\theta )\geq \frac{1}{\gamma^{M}}].$$

Here, I[c] returns 1 if some condition *c* is true; otherwise, I[c] returns 0. Given a hyper-parameter $\gamma^{M}$, our proposed metric σ(θ) estimates the stochastic similarity between every two RNA homologs without aligning them. Our proposed metric σ(θ) considers only nucleotide matches (*i*, *k*) satisfying the condition $p_{ik}^{M}(\theta )\geq \frac{1}{\gamma^{M}}$ to be eligible.

We extend Equation (1) into a generalized predictive recursion for multiple SAF, replacing pairwise nucleotide matching probabilities $p_{ik}^{M}(\theta )$ with alignment column matching probabilities and replacing SAF-based average base-pairing probabilities [or “consistency” (Do et al. 2005; Tagashira and Asai 2022)] $p_{ij}^{P}(\theta ),p_{kl}^{P}(\theta )$ with alignment column base-pairing probabilities


$$p_{IK}^{M}(\theta ;X,Y)\overset{\text{def}}{=}\frac{1}{\left|X||Y\right|}\sum_{xy:x\in X,y\in Y}p_{I_{x}K_{y}}^{M}(\theta ;x,y)$$

$$\begin{array}{c} p_{IJ}^{P}(\theta ;X)\overset{\text{def}}{=}\frac{1}{\left|X\right|}\sum_{x:x\in X}p_{I_{x}J_{x}}^{P}(\theta ;x)\\ p_{KL}^{P}(\theta ;Y)\overset{\text{def}}{=}\frac{1}{\left|Y\right|}\sum_{y:y\in Y}p_{K_{y}L_{y}}^{P}(\theta ;y). \end{array}$$

Here, *X* and *Y* are every two aligned sets of RNA homolog sequences to be aligned. Moreover, I,J,K,L are every four alignment columns, and *I_x_* is any RNA homolog *x*’s nucleotide stored in *I*. $p_{ik}^{M}(\theta ;x,y)$ is the explicit form of each nucleotide matching probability $p_{ik}^{M}(\theta )$ regarding every two RNA homologs *x*, *y*; and $p_{ij}^{P}(\theta ;x)$ is the explicit form of each average base-pairing probability $p_{ij}^{P}(\theta )$ regarding *x*. We align and fold every two aligned sets keeping only their matching profiles until we reach the root of a guide tree from leaves.

#### 2.1.3 Auto-correction of SAF hyper-parameters

The disadvantage of *γ*-centroid estimators is that they rely on hyper-parameters (e.g. hyper-parameters $\gamma^{M},\gamma^{P}$ in our *γ*-centroid SAF). In practice, we have to determine hyper-parameters before performing *γ*-centroid estimators. This study proposes a reasonable way for determining hyper-parameters in performing *γ*-centroid SAF. The ‘sum-of-pairs score’ (SPS) of any predicted SAF candidate A against any possible multiple alignment A′ is regarding *C* as an SPS normalization factor (Thompson et al. 1999). Here, $M_{xy}(A)$ is a set of all matched nucleotide pairs between every two RNA homologs *x*, *y* in any SAF candidate A; $I_{xy}(A)$ is a set of all inserted nucleotides between *x*, *y* in A; $D_{xy}(A)$ is a set of all deleted nucleotides between *x*, *y* in A.

Theorem 1. *As a result, we can measure the approximate expected SPS of any predicted SAF candidate* A*against all possible multiple alignmentscomputing posterior indel probabilities*

$$\begin{array}{c} \sigma (A,A\prime )\overset{\text{def}}{=}\frac{1}{C}\sum_{xy:x<y}\{\sum_{ik:(i,k)\in M_{xy}(A)}I[(i,k)\in M_{xy}(A\prime )]\\ +\sum_{i:i\in I_{xy}(A)}I[i\in I_{xy}(A\prime )]+\sum_{k:k\in D_{xy}(A)}I[k\in D_{xy}(A\prime )]\} \end{array}$$

$$\begin{array}{c} E[\sigma (A,\cdot );\theta ]\overset{\text{def}}{=}\frac{1}{C}\sum_{xy:x<y}[\sum_{ik:(i,k)\in M_{xy}(A)}p_{ik}^{M}(\theta ;x,y)\\ +\sum_{i:i\in I_{xy}(A)}p_{i}^{I}(\theta ;x,y)+\sum_{k:k\in D_{xy}(A)}p_{k}^{D}(\theta ;x,y)]. \end{array}$$

$$\begin{array}{c} p_{i}^{I}(\theta ;x,y)\overset{\text{def}}{=}1-\sum_{k:1\leq k\leq M}p_{ik}^{M}(\theta ;x,y)\\ p_{k}^{D}(\theta ;x,y)\overset{\text{def}}{=}1-\sum_{i:1\leq i\leq N}p_{ik}^{M}(\theta ;x,y). \end{array}$$

Finally, we can optimize hyper-parameters $\gamma^{M},\gamma^{P}$ as well as predicted SAF by enumerating possible values of $\gamma^{M},\gamma^{P}$ as in Supplementary Algorithm S1. Our SAF hyper-parameter auto-correction is similar to hyper-parameter auto-correction used in folding single RNA sequences—called pseudo-expected accuracy optimization (Hamada et al. 2010; Sato and Kato 2022)—because both these methods optimize *γ*-centroid hyper-parameters based on posterior probabilities.

#### 2.1.4 Ali-folding post-process of predicted SAF

Folding RNA alignment—or ali-folding—is one of the most potent ways to find ‘conserved base pairings’ in RNA alignment as well as SAF (Bernhart et al. 2008; Seemann et al. 2008; Hamada et al. 2011; Tagashira and Asai 2022). As LocARNA adopts RNAalifold (Bernhart et al. 2008) for the ali-folding post-processor of LocARNA’s predicted SAF (Will et al. 2007), we adopt ConsAlifold (Tagashira and Asai 2022) as the ali-folding post-processor of our predicted SAF. ConsAlifold uses (i) ConsProb’s posterior probabilities based on SAF and (ii) RNAalifold’s posterior probabilities based on ali-folding to produce a final firm folded alignment. SAF is optimized based on the balance between alignment and folding (AF) of RNA homologs. Therefore, consensus structures formed by SAF are not always optimum given “fixed” alignment. ConsAlifold is appropriate as ConsAlign’s lightweight post-processor since ConsAlign does not perform the iterative (time-consuming) refinement of produced immature SAF after progressive SAF, like conventional SAF tools by default.

#### 2.1.5 Ensemble of different SAF scoring models

Unifying various stochastic models for a prediction—referred to as a model ensemble—has been utilized in folding single RNA sequences to achieve more accurate structure prediction (Aghaeepour and Hoos 2013; Singh et al. 2019). In our SAF prediction, we combine (i) Tagashira and Asai (2022)’s inside–outside algorithm based on Turner’s model (Turner and Mathews 2010) and (ii) Tagashira and Asai (2022)’s inside–outside algorithm reparameterized with ConsTrain’s learned scoring model to create a SAF scoring model ensemble. (We describe ConsTrain in Section 2.2.) We use averaged column-matching probabilities and averaged column base-pairing probabilities


$$p_{IK}^{M}(\theta^{\text{turner}},\theta^{*};X,Y)\overset{\text{def}}{=}\frac{1}{2}[p_{IK}^{M}(\theta^{\text{turner}};X,Y)+p_{IK}^{M}(\theta^{*};X,Y)]$$

$$p_{IJ}^{P}(\theta^{\text{turner}},\theta^{*};X)\overset{\text{def}}{=}\frac{1}{2}[p_{IJ}^{P}(\theta^{\text{turner}};X)+p_{IJ}^{P}(\theta^{*};X)].$$

Here, $\theta^{\text{turner}}$ represents the SAF scoring parameters used in Tagashira and Asai (2022), but sequence alignment scoring parameters are transplanted (or copied) from our trained scoring parameters $\theta^{*}$ into $\theta^{\text{turner}}$. This transplantation is available since two scoring parameter sets $\theta^{\text{turner}},\theta^{*}$ adopt the CONTRAlign model as their sequence alignment scoring. The difference between SAF scoring parameter sets $\theta^{\text{turner}}$ and $\theta^{*}$ is that they adopt Turner’s and the CONTRAfold models, respectively. We expect that parameters transplanted from our trained scoring parameters $\theta^{*}$ will improve the predictive ability of Turner’s model-based scoring parameters $\theta^{\text{turner}}$. ConsTrain enriches its SAF scoring parameters by trying to adjust their expected occurrences to observed occurrences, as CONTRAfold and CONTRAlign do (Do et al. 2006a, 2006b). Therefore, ConsTrain does not include thermodynamics as a principle. Relying on our proposed model ensemble, we improve ConsAlign’s performance by including thermodynamics in our SAF prediction. We refer to ConsProb using our proposed model ensemble as ConsProb-ensemble. To distinguish ConsProb-ensemble, we refer to ConsProb based only on Turner’s model as ConsProb-Turner. Likewise, we refer to ConsProb reparameterized with ConsTrain’s learned scoring model as ConsProb-trained. We implemented our AF tool explained above as the ConsAlign version 0.1.7 (https://github.com/heartsh/consalign).

### 2.2 Computing SAF scoring parameters’ gradient

We can optimize the scoring parameters of each CLLM (including our SAF pair-CLLM), given their cost functions (Do et al. 2006a; Andronescu et al. 2007). We extract empirical parameter occurrence counts from observed pairwise AF examples and expected parameter occurrence counts from observed RNA sequence pairs. Combining these parameter occurrence counts, we can derive the ‘gradient’ of a pair-CLLM cost function. We compute the approximate expected counts of scoring parameters appearing in training RNA sequence pairs by conducting our inside-outside algorithm on sparse pairwise SAF iteratively.

#### 2.2.1 Forming the cost of training pairwise AF examples

We derive a ‘convex’ pair-CLLM cost function fully parameterized with tunable SAF scoring parameters from training homolog sequence pairs and pairwise AF examples. We score any pairwise AF example A by a simple inner product given any scoring parameter vector θ in the real *F*-dimensional space. [*F* is the number of features in any feature vector ϕ(A).] Here, a function ϕ(A) maps any pairwise AF example A to the feature vector that counts the occurrence of each *f*-th scoring parameter $\theta_{f}:(\theta_{f})\equiv \theta ,f\in \{1,\ldots ,F\}$ in A. Our feature vector ϕ(A) counts nucleotide matches/indels, their transitions, and fundamental features to be captured in RNA secondary structures (e.g. hairpin loop length and base pair stacking). [We describe the specific settings of ϕ(A) in Section 2.4.] This study assumes that any AF candidate A does not contain any pseudoknots (Sato et al. 2011; Jabbari et al. 2018) to simplify our proposed algorithms; we also do not consider any base-pairing indels, i.e. we must match each base-pairing in one RNA secondary structure of A with some base-pairing in the other RNA secondary structure of A, and vice versa, as implemented in many SAF tools (Hofacker et al. 2004; Kiryu et al. 2007; Will et al. 2007; Do et al. 2008; Sato et al. 2012). The pairwise SAF scoring s(A;θ) obeys an underlying pair-CLLM. As a result, each SAF scoring parameter *θ_f_* does not have to take the form of a log probability nor correspond only to a transition/emission (Do et al. 2006a).


$$s(A;\theta )\overset{\text{def}}{=}\theta^{T}\cdot \phi (A)$$

For the training AF example $A_{d}$ of the *d*-th training sequence pair, we prepare a “Boltzmann (probability) distribution”


$$A_{d}∼p_{d}(A_{d};\theta )\overset{\text{def}}{=}\frac{e^{s(A_{d};\theta )}}{Z_{d}(\theta )}$$

presuming any SAF ‘partition function’


$$Z_{d}(\theta )\overset{\text{def}}{=}\sum_{A^{\prime }:A^{\prime }\in A_{d}}e^{s(A^{\prime };\theta )}.$$

($A_{d}$ is the SAF ‘Boltzmann ensemble’—i.e. a set of all possible SAF candidates—of the *d*-th training sequence pair.) We obtain a convex pair-CLLM cost onto any training dataset *D* comprising each training sequence pair and each training AF example (Do et al. 2006a; Andronescu et al. 2007)

Theorem 2. *Our convex pair-CLLM cost* c(D;θ)*holds its gradientderived from any expected count vector*

$$c(D;\theta )\overset{\text{def}}{=}-\sum_{d:d\in \{1,\ldots ,|D|\}}\ln p_{d}(A_{d};\theta )\equiv \sum_{d}[\ln Z_{d}(\theta )-s(A_{d};\theta )].$$

$$\nabla c(D;\theta )\overset{\text{def}}{=}(\frac{\partial c(D;\theta )}{\partial \theta_{f}})\equiv \sum_{d:d\in \{1,\ldots ,|D|\}}\{E_{d}[\phi (\cdot );\theta ]-\phi (A_{d})\}$$

$$E_{d}[\phi (\cdot );\theta ]\overset{\text{def}}{=}\sum_{A\prime :A\prime \in A_{d}}p_{d}(A\prime ;\theta )\phi (A\prime ).$$

We extend our sparse inside–outside algorithm to estimate an approximate amount of any expected count vector $E_{d}[\phi (\cdot );\theta ]$ (Supplementary Section S1) replacing any SAF Boltzmann distribution $p_{d}(A\prime ;\theta )$ with $p_{d}^{S}(A\prime ;\theta )$, a sparse version of $p_{d}(A\prime ;\theta )$:


$$E_{d}[\phi (\cdot );\theta ]\equiv \sum_{A\prime :A\prime \in A_{d}}p_{d}(A\prime ;\theta )\phi (A\prime )\approx \sum_{A\prime :A\prime \in A_{d}^{S}}p_{d}^{S}(A\prime ;\theta )\phi (A\prime )$$

$$\begin{array}{c} A\prime ∼p_{d}(A\prime ;\theta )\approx p_{d}^{S}(A\prime ;\theta )\overset{\text{def}}{=}\frac{e^{s(A^{\prime };\theta )}}{Z_{d}^{S}(\theta )}\\ Z_{d}(\theta )\approx Z_{d}^{S}(\theta )\overset{\text{def}}{=}\sum_{A\prime :A\prime \in A_{d}^{S}}e^{s(A^{\prime };\theta )}. \end{array}$$

This approximated vector estimation does not change the running time $O(L^{2})$ and memory usage $O(L^{2})$ of our sparse inside–outside algorithm. (*L* is the longer length of the *d*-th training sequence pair.)

### 2.3 Optimizing SAF scoring parameters

Our parameter optimization goal is to find SAF scoring parameters that minimize their convex pair-CLLM cost. We can utilize general gradient-based optimization methods to complete our scoring parameter optimization. We aim to compute an optimized parameter vector


$$\begin{array}{c} \theta^{*}\overset{\text{def}}{=}\text{arg}\;\underset{\theta \in R^{F}}{\text{min}}c^{S}(D;\theta )\\ c^{S}(D;\theta )\overset{\text{def}}{=}\sum_{d:d\in \{1,\ldots ,|D|\}}[\ln Z_{d}^{S}(\theta )-s(A_{d};\theta )] \end{array}$$

(where $R^{F}$ is the real *F*-dimensional space) using the gradient of an approximated pair-CLLM cost $c^{S}(D;\theta )$


$$\begin{array}{c} \nabla c^{S}(D;\theta )\overset{\text{def}}{=}\sum_{d:d\in \{1,\ldots ,|D|\}}\{E_{d}^{S}[\phi (\cdot );\theta ]-\phi (A_{d})\}\\ E_{d}^{S}[\phi (\cdot );\theta ]\overset{\text{def}}{=}\sum_{A\prime :A\prime \in A_{d}^{S}}p_{d}^{S}(A\prime ;\theta )\phi (A\prime ). \end{array}$$

By Supplementary Algorithm S2, we can conduct the above convex parameter optimization iteratively. Supplementary Algorithm S2 is an instance of the Broyden–Fletcher–Goldfarb–Shanno (BFGS) algorithm, a quasi-Newton optimization algorithm widely used (Fletcher 1987).

Our approximated pair-CLLM cost $c^{S}(D;\theta )$ does not retain any regularization term. In other words, an optimized parameter vector $\theta^{*}$ is prone to be overfitted for each training AF dataset *D*. Thus, we minimize an L2-regularized non-convex cost instead of our unregularized convex cost $c^{S}(D;\theta )$: taking the two gamma distribution parameters *α*, *β* and the parameter group *G*(*f*) where each SAF scoring parameter *θ_f_* belongs. (We show *G*(*f*)’s settings in Section 2.4.) Supplementary Algorithm S3 based on Supplementary Algorithm S2 implements the above non-convex parameter optimization iteratively. Supplementary Algorithm S3 is an example of ‘majorization–minimization algorithms’, iterative optimization algorithms that minimize some convex upper bound of any non-convex cost function (Lange et al. 2000). Supplementary Algorithm S3 groups each SAF scoring parameter *θ_f_* into a cluster and penalizes each scoring parameter cluster by a shared regularization constant (Foo et al. 2009). The merit of introducing our adaptive regularization term $\frac{1}{2}\lambda^{T}\cdot (\theta_{f}^{2})$ is that we do not have to predetermine the value of our L2-regularization constant vector λ (e.g. by a grid search upon a validation AF dataset) since we (in addition to our SAF scoring parameters θ to be optimized) optimize λ in the same procedure (Foo et al. 2009). We implemented Supplementary Algorithm S3 as the ConsTrain version 0.1.7 (https://github.com/heartsh/consprob-trained).


$$\begin{array}{c} \theta^{*}\overset{\text{def}}{=}\text{arg}\;\underset{\theta \in R^{F}}{\text{min}}c^{R}(D;\theta ,\alpha ,\beta )\\ c^{R}(D;\theta ,\alpha ,\beta )\overset{\text{def}}{=}c^{S}(D;\theta )+\frac{1}{2}\lambda^{T}\cdot (\theta_{f}^{2})\\ \lambda \overset{\text{def}}{=}(\frac{\frac{\left|G(f)\right|}{2}+\alpha }{\beta +\frac{1}{2}\sum_{f\prime :f\prime \in \{1,\ldots ,F\}|G(f\prime )=G(f)}\theta_{f\prime }^{2}}) \end{array}$$

### 2.4 Configuring SAF parameters to be trained

Borrowing knowledge from conventional scoring parameter optimizations, we specify the concrete settings of our SAF parameters to be trained. We specify all the groups (or types) of our SAF parameters, as described in Section S2. The ‘CONTRAfold model’ is a CLLM that (from folded single RNA sequences) learns secondary structure scoring parameters based on RNA loop structures (Do et al. 2006a). The ‘CONTRAlign model’ is a pair-conditional random field (Lafferty et al. 2001; Sato and Sakakibara 2005) that trains sequence alignment scoring parameters from each sequence-aligned RNA pair (Do et al. 2006b). (Conditional random fields are a specialized instance of CLLMs where Boltzmann distributions are defined according to graphical models.) Our pair-CLLM inherits scoring parameter formats from both the CONTRAfold and the CONTRAlign models.

### 2.5 Benchmarking data

We used the Rfam version 14.4 (Kalvari et al. 2018) for our SAF parameter training and software benchmarking test. From Rfam, we collected 3129 aligned, folded families as the dataset “origin.” The seed reference AF of each collected RNA family retained at most 500 columns and 20 sequences. We randomly split the dataset “origin” into both the equal datasets “train origin” and “Rfam test.” From the dataset “train origin,” we collected each RNA family whose seed reference consensus structure is not any prediction product [e.g. neither by Pfold (Knudsen and Hein 2003) nor by RNAalifold (Bernhart et al. 2008)] as the dataset “train pure.” We random-sampled 10 RNA sequences from each RNA family holding at least 10 RNA sequences in the dataset “train pure.” Then, we retrieved the all-to-all pairwise sub-AF maps of these 10 RNA sequences, finally obtaining the dataset “Rfam train.” This random sequence sampling prevents our SAF parameter learning from overfitting due to the overwhelming number of RNA homologs belonging to the same large RNA family (Do et al. 2006a, 2008).

As a result, (i) the dataset “Rfam train” is composed of 2250 pairwise AF examples, and (ii) the dataset “Rfam test” is composed of 1565 multiple AF examples. We used the dataset “Rfam train” to learn ConsTrain. We used the dataset “Rfam test” to assess the performances of each benchmarked prediction method. Any entry in the dataset “Rfam train” and any entry in the dataset “Rfam test” are not derived from the same family to prevent structural homology from leaking between training data and test data (Rivas et al. 2012; Sato et al. 2021).

As another test data source, we adopted reference AF examples registered in RNAStralign (Tan et al. 2017). We split each complete AF example comprising all RNA sequences into the sub-AF of every 20 or fewer RNAs. At last, we obtained the dataset “RNAStralign processed,” composed of 923 multiple AF examples. From the BRAliBase II (Gardner et al. 2005), we also downloaded dataset 1, containing reference RNA sequence alignments, as a test dataset.

**Figure 3.**
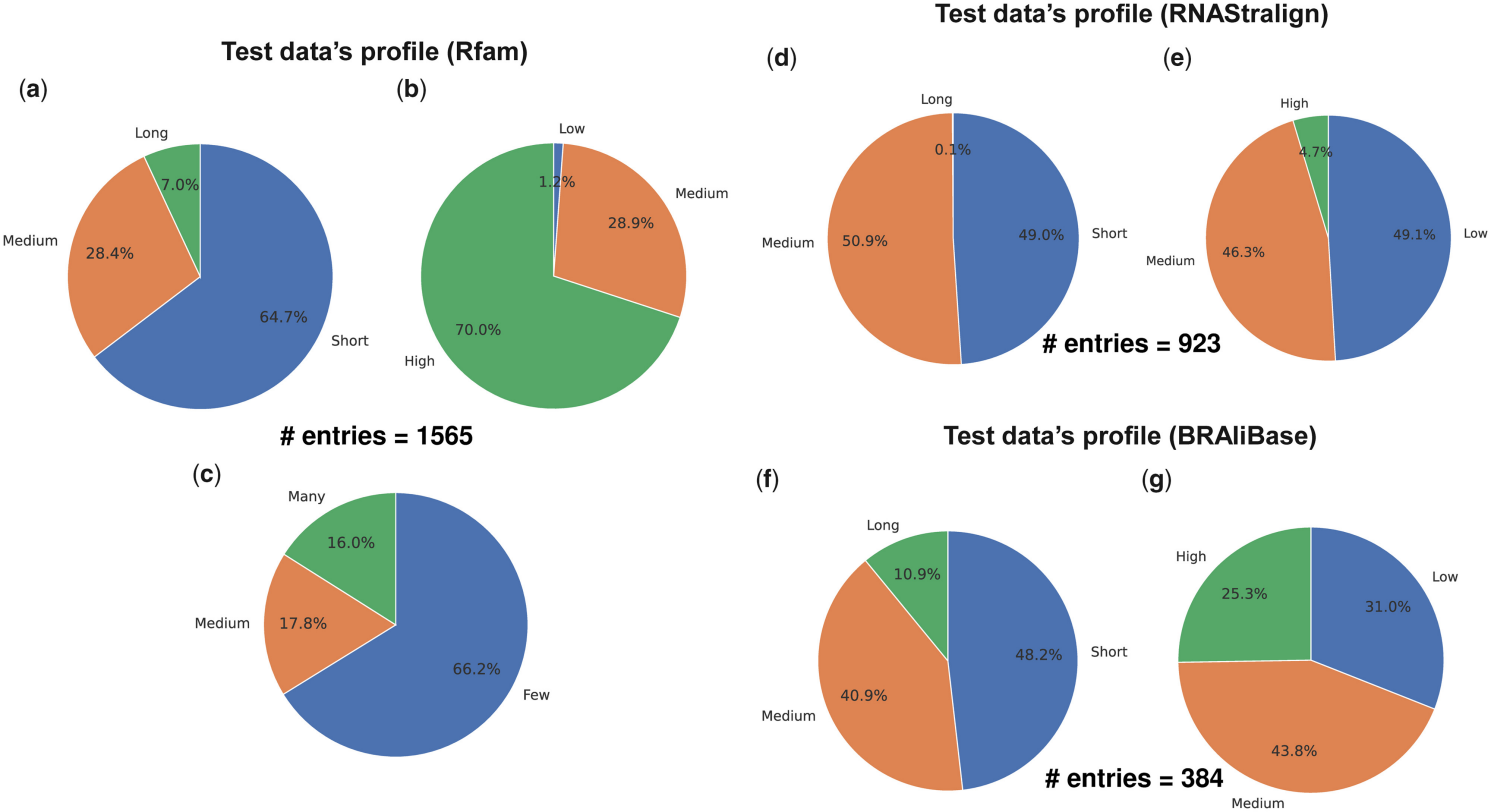
Summarized profile of each test alignment dataset regarding (a, d, f) average RNA sequence lengths, (b, e, g) pairwise sequence identities, and (c) the numbers of RNAs (available only for the dataset “Rfam test”). (Short) Average RNA sequence lengths are shorter than 100. (Long) Average RNA sequence lengths are at least 200. (Low) Pairwise sequence identity is ˂0.6. (High) Pairwise sequence identity is at least 0.8. (Few) The numbers of RNA sequences are fewer than six. (Many) The numbers of RNA sequences are at least 11. (Medium) The remaining part of each pie chart is covered. A pie chart regarding the number of RNA sequences was unavailable for the dataset “RNAStralign processed” since the dataset “RNAStralign processed” has 20 RNA sequences in each of most test AF examples. Likewise, a pie chart regarding the numbers of RNA sequences was unavailable for BRAliBase’s dataset 1 since BRAliBase’s dataset 1 has five RNA sequences in each test alignment.

RNAStralign and BRAliBase contain alignments derived from Rfam. Therefore, we checked the homology of RNAStralign and BRAliBase to Rfam using Infernal, an RNA homology search tool (Nawrocki and Eddy 2013). More specifically, we built the profile of each test alignment in the dataset “RNAStralign processed” and BRAliBase’s dataset 1 using Infernal’s cmbuild. After cmbuild, we mapped each training pairwise alignment in the dataset “Rfam train” to each alignment profile using Infernal’s cmsearch. We did not perform Infernal’s cmcalibrate before cmsearch because cmcalibrate is time-consuming, and the dataset “RNAStralign processed” and BRAliBase’s dataset 1 are large. (cmcalibrate is essential if we wish to consider structural information in addition to sequential information upon Infernal.) As a result, only one alignment in each of the dataset “RNAStralign processed” and BRAliBase’s dataset 1 detected homology to the dataset “Rfam train.” We decided not to remove any alignments from the dataset “RNAStralign processed” and BRAliBase’s dataset 1. We summarized the profile of each test alignment dataset in Fig. 3.

### 2.6 Conventional AF tools for experiments

Using default command line arguments, we compared (i) the RAF version 1.00, (ii) the LocARNA version 2.0.0, (iii) the SPARSE version 2.0.0 (Will et al. 2015), (iv) the DAFS version 0.0.3 (Sato et al. 2012), and (v) the LinearTurboFold version 1 (Li et al. 2021) to ConsAlign. We chose these conventional AF tools because they take at most quadratic time complexity and because we use large-scale test datasets. Each conventional tool’s characteristics are described in Supplementary Section S3.

By default, DAFS turns off its pseudoknot-aware prediction based on IPknot (Sato et al. 2011). We included RAF in our tool comparison as a recent SAF tool depending on trained SAF parameters. RAF scores potential SAF using the posterior probabilities of folding single RNA sequences and those of pairwise sequence alignment. In contrast, ConsAlign uses the posterior probabilities of sparse pairwise SAF. More specifically, RAF obtains the score of any pairwise SAF candidate A using RAF’s weights $w_{x}^{M},w_{y}^{P}$ to be trained. Here, M(A) is a set of all matched nucleotide pairs in any pairwise SAF candidate A, and $M^{P}(A)$ is a set of all matched base-pairing nucleotide quadruples in A. Moreover, a function $\phi_{ik}^{M}(x)$ maps any nucleotide pair (*i*, *k*) into the *x*-th basis feature based on CONTRAlign’s posterior matching probabilities. Likewise, a function $\phi_{ij}^{P}(y)$ maps any nucleotide pair (*i*, *j*) into the *y*-th basis feature based on CONTRAfold’s posterior base-pairing probabilities. RAF trains its weights $w_{x}^{M},w_{y}^{P}$ from training RNA sequence pairs and training pairwise AF examples based on max-margin optimization [also known as the structured support vector machine (Tsochantaridis et al. 2005)]. As we can see, RAF trains its weights $w_{x}^{M},w_{y}^{P}$ to posterior probability-based basis features $\phi_{ik}^{M}(x),\phi_{ij}^{P}(y)$. In contrast, ConsAlign trains SAF scoring parameters θ for deriving sparse posterior probabilities $p_{ik}^{M}(\theta ),p_{ij}^{P}(\theta )$. Conventional AF tools optimized their parameters based on reference data. For example, RAF conducted its max-margin optimization using Rfam. As another example, LinearTurboFold trained its parameters using RNAStralign (Harmanci et al. 2011; Tan et al. 2017; Li et al. 2021). We retrained only RAF using the dataset “Rfam train” due to experimental cost. We also do not report comparative results regarding ConsAlign based on cross-validation since we use external test alignment datasets to Rfam—RNAStralign and BRAliBase. We do not perform the experimental comparison between ConsAlign and Dowell and Eddy (2006)’s pair-SCFG (http://eddylab.org/software/consan) because the latter predicts only pairwise SAF and because predicting multiple SAF is a more general situation than pairwise SAF.


$$\begin{array}{c} \sum_{ik:(i,k)\in M(A)}\sum_{x:x\in \{1,\ldots ,4\}}w_{x}^{M}\phi_{ik}^{M}(x)\\ +\sum_{\mathrm{ijkl}:(i,j,k,l)\in M^{P}(A)}\sum_{y:y\in \{1,\ldots ,4\}}w_{y}^{P}[\phi_{ij}^{P}(y)+\phi_{kl}^{P}(y)] \end{array}$$

### 2.7 AF prediction accuracy metrics

Across all test AF examples, we find the numbers of true positives, true negatives, false positives, and false negatives TP, TN, FP, FN regarding structure quality while benchmarking each tool. We compute an *F*1 score and a Matthews correlation coefficient (MCC) in each test AF dataset:


$$\begin{array}{c} F1\overset{\text{def}}{=}\frac{2\times \text{TP}}{2\times \text{TP}+\text{FP}+\text{FN}}\\ \text{MCC}\overset{\text{def}}{=}\frac{\text{TP}\times \text{TN}-\text{FP}\times \text{FN}}{\sqrt{\left(\text{TP}+\text{FP})(\text{TP}+\text{FN})(\text{TN}+\text{FP})(\text{TN}+\text{FN}\right)}}. \end{array}$$

We obtain both the SPS and the structure conservation index (SCI) of each predicted AF product to measure its matching accuracy. Each SCI is defined as given the minimum free energy $E^{\text{align}}$ of each input alignment and the average $\bar{E}$ of each sequence’s minimum energy (Washietl et al. 2005). Each SCI close to 1 indicates the structural conservation of each input alignment. Furthermore, each SCI ˃1 proves that compensatory and/or consistent mutations support each alignment’s structural conservation via covariance scores. Each SCI quantifies the structural conservation of a corresponding RNA alignment from a thermodynamic viewpoint and, therefore, diagnoses the matching quality of each predicted RNA alignment considering potential structure quality. Will et al. (2012) reported that the SCI and the SPS of each alignment produced by LocARNA-P did not correlate well in Rfam.


$$E^{\text{align}}/\bar{E}$$

### 2.8 Implementations and benchmark environments

Employing multi-threading to provide a more comfortable user experience, we implemented ConsTrain and ConsAlign in Rust (https://www.rust-lang.org). ConsTrain used the two gamma distribution parameters α←0,β←1 proposed by Foo et al. (2009). In ConsTrain, we implemented two different initialization methods for SAF scoring parameters to be trained. (i) In our first initialization method, corresponding scoring parameter values were transferred from the CONTRAfold and the CONTRAlign models. (ii) In the other initialization method, SAF scoring parameters were initialized by random values derived from the normal distribution with both the mean 0 and the variance $\frac{1}{F}$. ConsAlign used the four lower/upper bounds $i^{\text{min}}\leftarrow 0,i^{\text{max}}\leftarrow 7,j^{\text{min}}\leftarrow 0,j^{\text{max}}\leftarrow 3$ found in Supplementary Algorithm S1. Also, ConsAlifold performed in ConsAlign took the *γ*-centroid hyper-parameter $2^{0}+1=2$ because this *γ*-centroid hyper-parameter value showed the highest structure prediction accuracy among the possible *γ*-centroid hyper-parameter values $2^{-4}+1,\ldots ,2^{10}+1$ in Tagashira and Asai (2022). For our prediction software benchmarks, we used a workstation loading AMD Ryzen Threadripper PRO 3955WX with 32GB of RAM, 32 threads, and a clock rate of 3.9 GHz.

## 3 Results

### 3.1 Benchmarking ConsAlign to conventional methods

**Figure 4.**
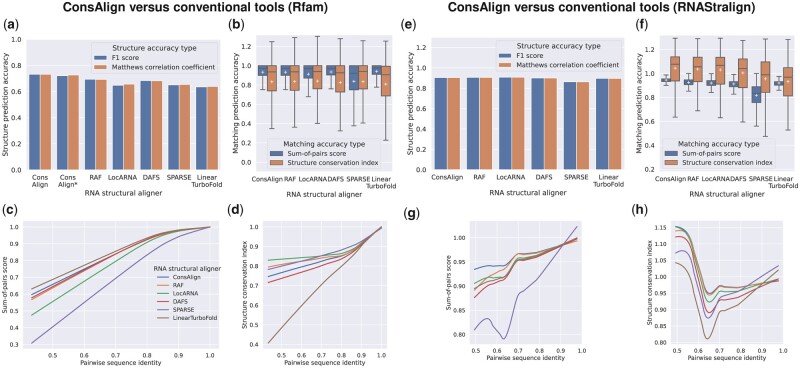
AF prediction accuracy comparison among ConsAlign and conventional AF tools. (a, e) Methods were compared in terms of F1 scores and MCCs. (b, f) Each method’s distributions of SPSs and SCIs are visualized in box plots. Each tool’s (c, g) SPSs and (d, h) SCIs are shown in lowess curves (Cleveland 1981) to the pairwise sequence identity of reference alignments. Outliers were omitted from box plots. Each white cross indicates a corresponding method’s average. The datasets (a–d) “Rfam test” and (e–h) “RNAStralign processed” were used. ConsAlign was based on ConsProb-ensemble and our proposed transfer learning. (ConsAlign*) ConsAlign disabled its RNAalifold subroutine from producing alignment column base-pairing probabilities (Bernhart et al. 2008) to check ConsAlign’s pure prediction performance.

In the dataset “Rfam test,” ConsAlign consistently performed better structure prediction accuracy than the other AF tools, whereas LinearTurboFold (Li et al. 2021) had the lowest rank regarding secondary structure prediction accuracy (Fig. 4a). ConsAlign showed comparable SPSs among state-of-the-art AF tools, while SPARSE (Will et al. 2015) indicated the poorest SPSs (Fig. 4b and c; Supplementary Table S2). Regarding SCIs, all AF tools, including ConsAlign, were competitive, except for LinearTurboFold, which does not perform as well (Fig. 4b and d; Supplementary Table S2). In Supplementary Tables S2–S4, we reported ConsAlign’s two-sided, paired *t*-tests to conventional AF tools upon each test alignment dataset, including statistical significance.

When we switched our test dataset from the dataset “Rfam test” to the dataset “RNAStralign processed,” ConsAlign competed with most AF tools regarding predicted structures’ quality, separating SPARSE from the others (Fig. 4e). ConsAlign was superior to conventional AF tools from the viewpoint of SPSs, and SPARSE suffered lower SPSs compared to the others (Fig. 4f and g; Supplementary Table S3). ConsAlign was comparable to RAF (Do et al. 2008) and LocARNA (Will et al. 2007) in SCIs (Fig. 4f and h; Supplementary Table S3). Surprisingly, LinearTurboFold was more disadvantageous to SCIs than the others, albeit performing moderate SPSs (Fig. 4f–h).

**Figure 5.**
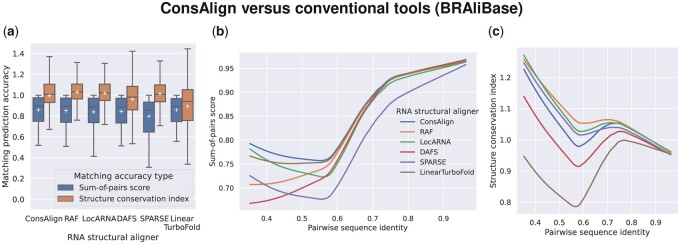
Predicted AF products’ quality among modern AF tools. (a) Each method’s SPSs and SCIs are visualized in box plots. Each method’s (b) SPSs and (c) SCIs are expressed in lowess curves to the pairwise sequence identity of reference alignments. BRAliBase’s dataset 1 was used.

**Figure 6.**
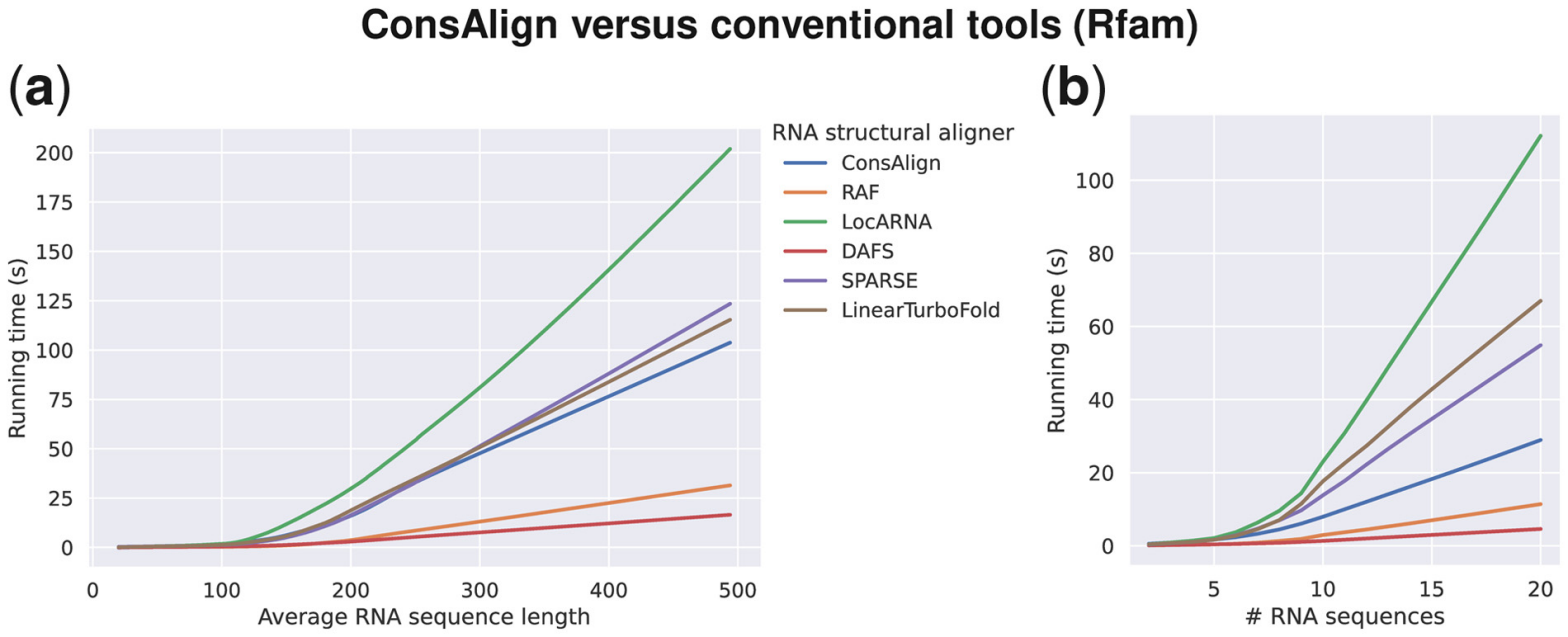
Running times of benchmarked AF tools in lowess curves to (a) average RNA sequence lengths and (b) the numbers of RNA sequences. The dataset “Rfam test” was used. ConsAlign was based on ConsProb-ensemble and our proposed transfer learning. For fairness, ConsAlign’s multi-threading was turned off.

In BRAliBase’s dataset 1 (Gardner et al. 2005), ConsAlign and the existing AF tools other than SPARSE were almost equally precise regarding SPSs (Fig. 5a and b; Supplementary Table S4). However, SPARSE’s predicted AF was graded highly together with ConsAlign, RAF, and LocARNA regarding SCIs (Fig. 5a and c; Supplementary Table S4). In the dataset “Rfam test,” lowess curves were smoother than the other test alignment datasets (Figs. 4 and 5) since most of its test SAFs had at least 60% pairwise sequence identity (Fig. 3). In each test alignment dataset, ConsAlign took the median prediction running time among available AF tools (Fig. 6; Supplementary Figs. S3 and S4).

### 3.2 Verifying our proposed training

**Figure 7.**
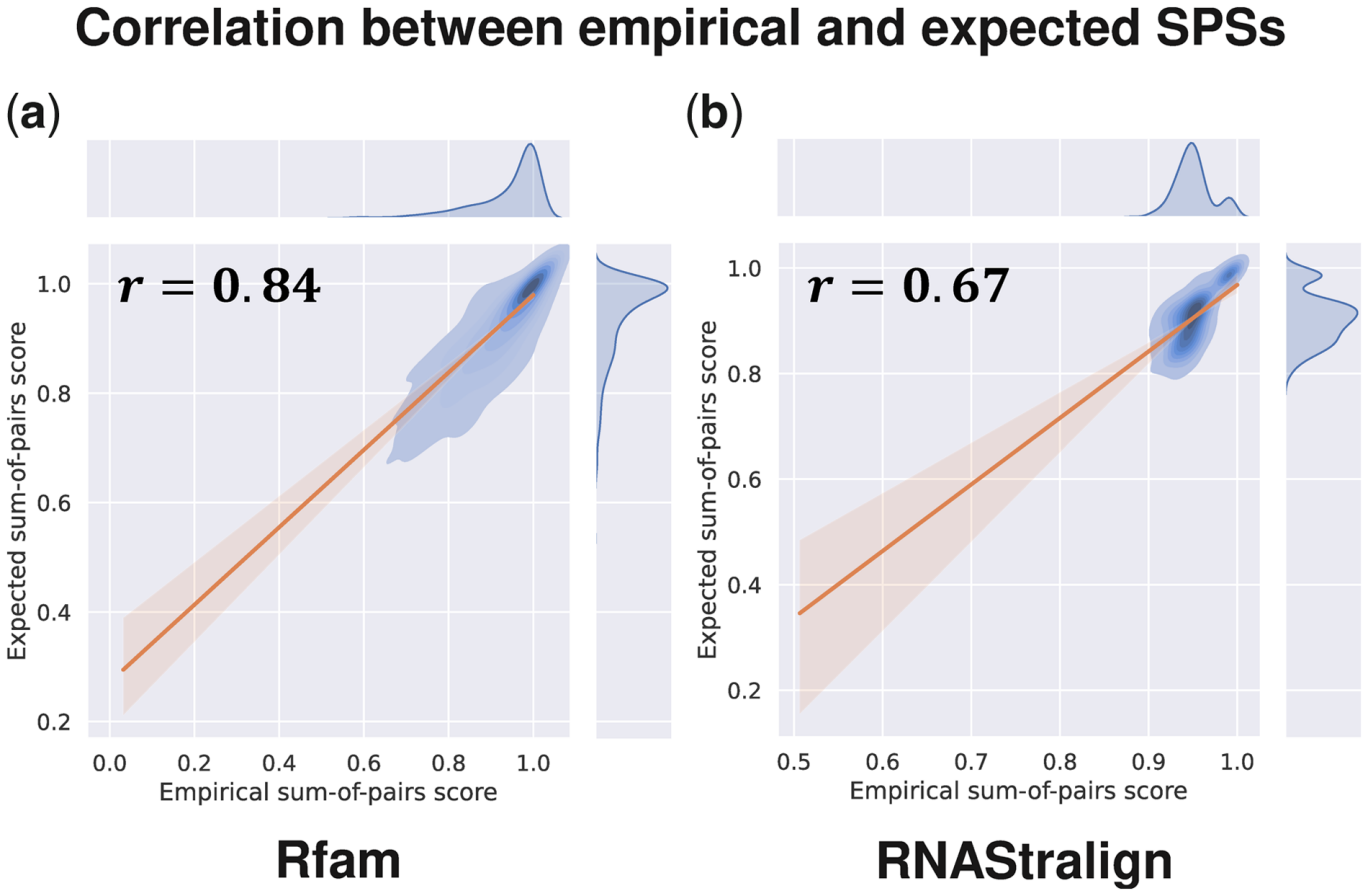
Contrast between ConsAlign’s empirical and expected SPSs in the datasets (a) “Rfam test” and (b) “RNAStralign processed.” (*r*) Each Pearson correlation coefficient was noted. ConsAlign was based on ConsProb-ensemble and our proposed transfer learning.

**Figure 8.**
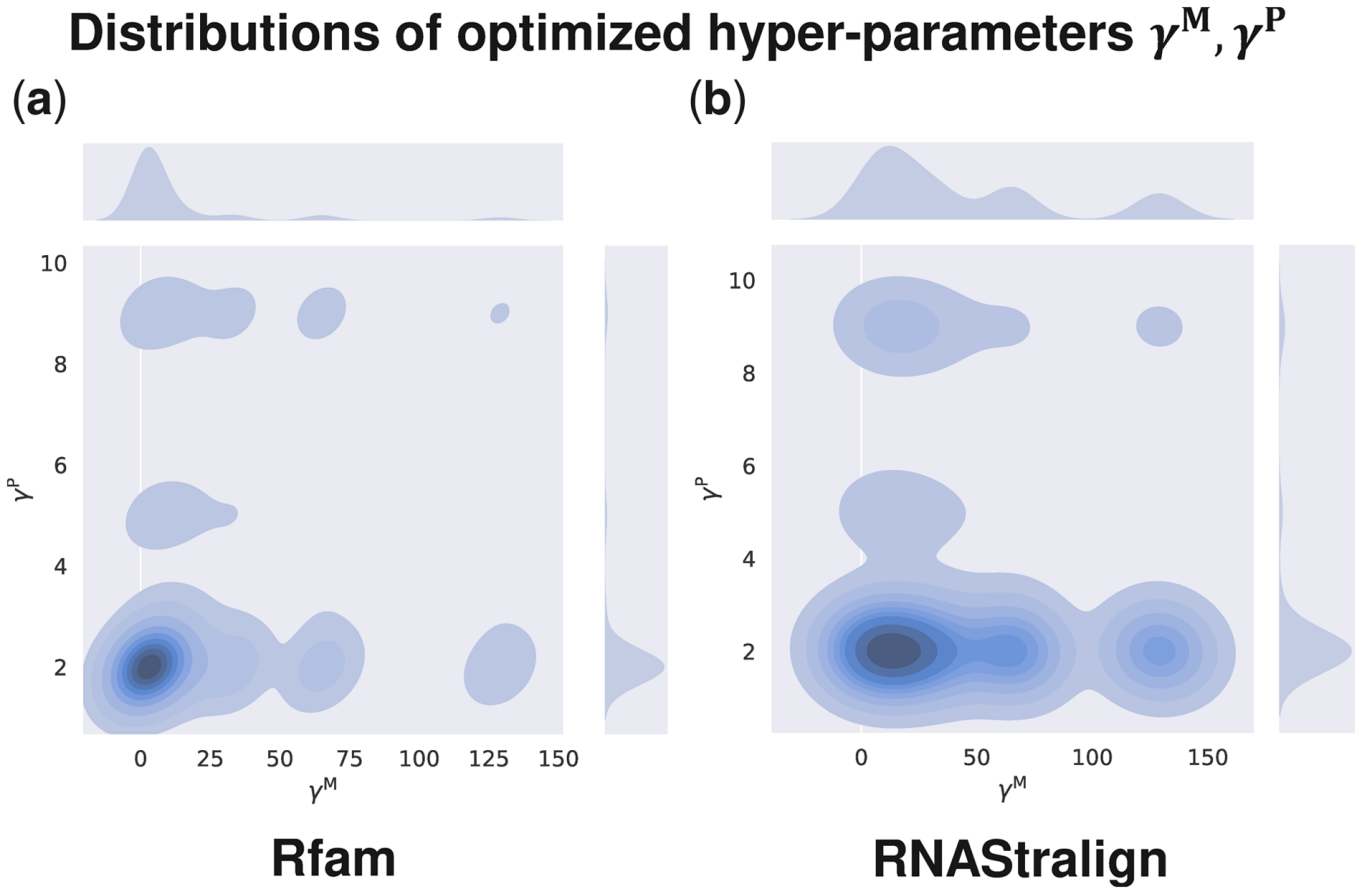
Distributions of ConsAlign’s optimized hyper-parameters $\gamma^{M},\gamma^{P}$ in the datasets (a) “Rfam test” and (b) “RNAStralign processed.” ConsAlign was based on ConsProb-ensemble and our proposed transfer learning.

ConsTrain took only about 30 epochs when using our proposed transfer learning (Supplementary Fig. S5). On the other hand, ConsTrain consumed around 90 epochs when we initialized our SAF scoring parameters to random values (Supplementary Fig. S5). ConsTrain continuously reduced our L2-regularized non-convex cost in both parameter initialization methods (Supplementary Fig. S5a), boosting average expected SPSs across the dataset “Rfam train” (Supplementary Fig. S5b). In each test dataset, ConsAlign’s expected SPSs highly correlated with its observed SPSs (Fig. 7; Supplementary Fig. S6a), and ConsAlign’s tuned hyper-parameters were principally distributed around lower bounds (Fig. 8; Supplementary Fig. S6b).

**Figure 9.**
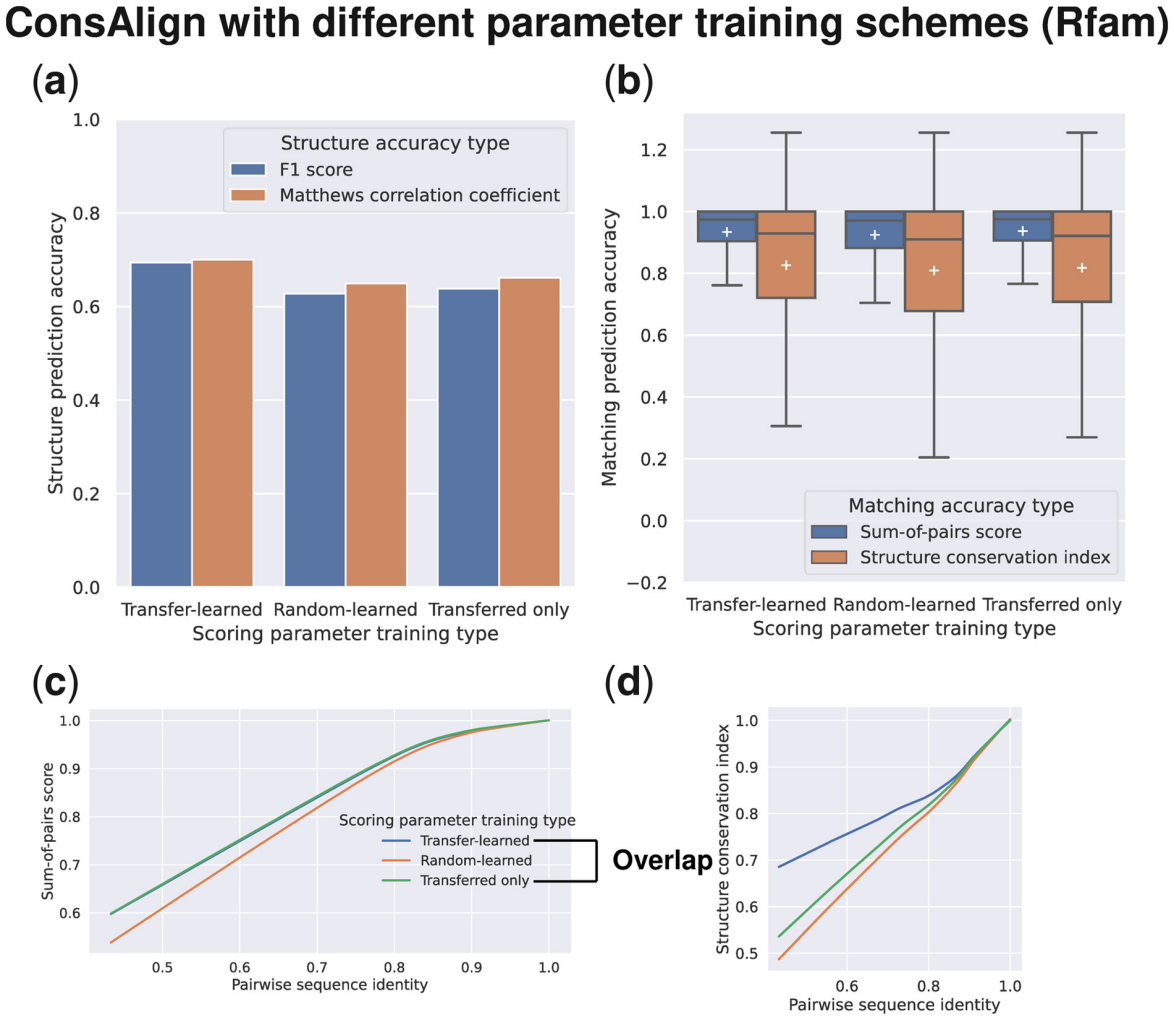
AF prediction accuracy comparison among the different parameter training schemes of ConsTrain. (Transfer-learned) SAF scoring parameters were transfer-learned. (Random-learned) Random values initialized SAF scoring parameters before training. (Transferred only) SAF scoring parameters were transferred but then were not trained. This figure’s configuration is the same as Fig. 4. The dataset “Rfam test” was used. ConsAlign was based on ConsProb-trained (Tagashira and Asai 2022) alone and disabled an RNAalifold subroutine.

In the dataset “Rfam test,” ConsTrain with our proposed transfer learning demonstrated the best structure prediction accuracy among available parameter training schemes (Fig. 9a). Demonstrated parameter training schemes showed the same level of SPSs (Fig. 9b and c). However, our proposed transfer learning distanced the others regarding SCIs (Fig. 9b and d). Shifting to the dataset “RNAStralign processed,” ConsAlign was enhanced at structural prediction accuracy when we focused on our transfer learning against our scoring parameters that were only transferred (Supplementary Fig. S7). From BRAliBase’s dataset 1, predicted matches’ quality was raised in ConsAlign when we compared our transfer learning with the others (Fig. 11a and b; Supplementary Fig. S9a).

### 3.3 Verifying our proposed model ensemble

**Figure 10.**
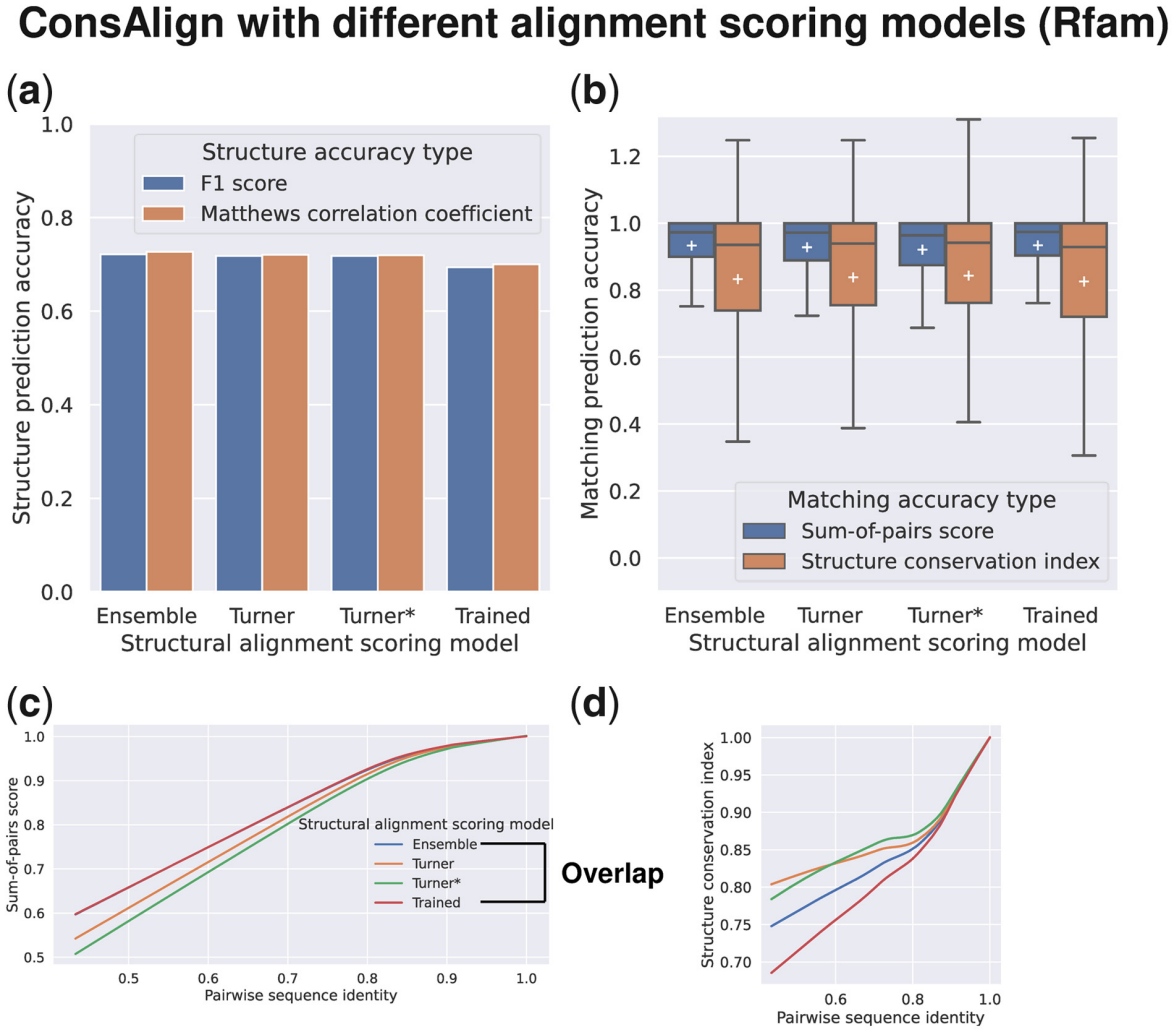
AF prediction accuracy comparison among the different SAF scoring models of ConsAlign. (Ensemble) ConsProb-ensemble was used. (Turner) Only ConsProb-Turner was used as a posterior probability inference method. (Turner*) Learned sequence alignment parameters were not transplanted into ConsProb-Turner to check their performance in it. (Trained) Only ConsProb-trained was used as a posterior probability inference method. This figure’s configuration is the same as Fig. 4. The dataset “Rfam test” was used. ConsAlign was based on our proposed transfer learning and disabled an RNAalifold subroutine.

**Figure 11.**
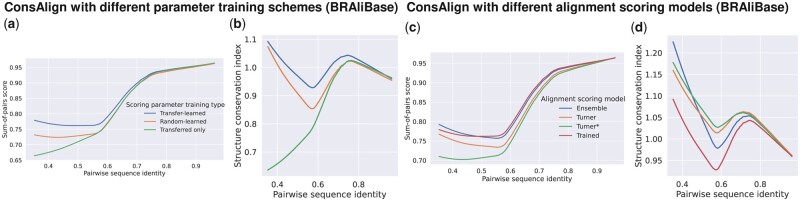
AF prediction accuracy comparison among (a, b) the different parameter training schemes and (c, d) the different SAF scoring models of ConsAlign. This figure’s configuration is the same as Figs. 9 and 10. BRAliBase’s dataset 1 was used.

In the dataset “Rfam test,” ConsProb-ensemble drew advantages from ConsProb-Turner, which outperformed ConsProb-trained, in structural accuracy (Fig. 10a). In contrast, ConsProb-trained as well as ConsProb-ensemble performed better than ConsProb-Turner in SPSs (Fig. 10b and c). Regarding SCIs, ConsProb-Turner ameliorated ConsProb-trained used in ConsProb-ensemble (Fig. 10b and d). Regarding structural quality and SPSs, ConsProb-ensemble capitalized on ConsProb-trained, which surpassed ConsProb-Turner, using the dataset “RNAStralign processed” (Supplementary Fig. S8a–c). ConsProb-Turner did not boost the SCIs of ConsProb-trained in ConsProb-ensemble (Supplementary Fig. S8b and d). The SPSs of ConsProb-ensemble did not improve referring to ConsProb-trained upon BRAliBase’s dataset 1 (Fig. 11c; Supplementary Fig. S9b). Interestingly, the SCIs of ConsProb-ensemble rose with the help of ConsProb-Turner compared to ConsProb-trained, especially in the range of lower sequence identity (Fig. 11d; Supplementary Fig. S9b).

In each test dataset, transplanting trained scoring parameters improved ConsAlign’s SPSs compared to the case where this transplantation was unavailable (Figs. 10b and c and 11c; Supplementary Figs. S8b and c and S9b). However, CONTRAlign’s original trained parameters (Do et al. 2006b) were slightly more effective rather than transplanted sequence alignment parameters when we measured ConsAlign’s SCIs using the dataset “RNAStralign processed” and BRAliBase’s dataset 1 (Supplementary Figs. S8b and d and S9b; Fig. 11d).

## 4 Conclusion and discussion

We invented ConsTrain—an efficient gradient-based learning method of SAF scoring parameters—and applied it to ConsAlign—a model ensemble-based AF tool. ConsAlign attained competitive AF quality, especially structural accuracy and SPSs, in our AF tool benchmarks. In our AF software comparison, LinearTurboFold (Li et al. 2021) and SPARSE (Will et al. 2015) were the weakest in SCIs and SPSs, respectively. LinearTurboFold is an iterative method different from the other benchmarked simultaneous methods. Therefore, we can interpret that simultaneous solutions enhanced obtained matches’ quality by the more direct transmission of structural conservation among homologs than iterative solutions. However, SCIs do not guarantee that secondary structures derived from alignments with low SCIs also have low accuracy, as LinearTurboFold performed. SCIs are mere metrics based on “minimum free energy,” and minimizing free energy does not always ensure that obtained secondary structures’ accuracy is maximized, as *γ*-centroid estimators (including ConsAlign) demonstrated (Ding et al. 2005; Hamada et al. 2009a, 2009b, 2011).

SPARSE realizes its efficient computation by employing only structure-based constraints, whereas the other SAF tools—ConsAlign, LocARNA (Will et al. 2007), RAF (Do et al. 2008), and DAFS (Sato et al. 2012)—employ matching-based constraints in addition to structure-based constraints. We can explain that leveraging both matching-based and structure-based constraints is more effective regarding obtained nucleotide matches’ quality than only structure-based constraints.

In each test alignment dataset, ConsAlign outperformed RAF regarding structural quality and SPSs. RAF conducts its SAF prediction using simple “non-simultaneous” posterior probabilities produced by CONTRAfold (Do et al. 2006a) and CONTRAlign (Do et al. 2006b). In contrast, ConsAlign predicts SAF using complex simultaneous posterior probabilities produced by ConsProb (Tagashira and Asai 2022). Digging deep, ConsAlign adopts ConsTrain’s simultaneous learning of posterior probabilities. RAF diverts CONTRAfold and CONTRAlign’s non-simultaneous learning of posterior probabilities, though RAF also trains simultaneous weights to these posterior probabilities in predicting SAF. In our conclusion, ConsAlign benefited more from ConsProb and ConsTrain’s complex “simultaneity” regarding AF accuracy compared to RAF.

ConsAlign showed competitive running time in each test alignment dataset. ConsAlign realized its good balance between SAF prediction accuracy/speed with stochastically possible SAF served by ConsProb and ConsTrain. ConsAlign introduced sparse dynamic programming and expected SPS-based hyper-parameter optimization, and they also contributed to this well performance balance.

ConsTrain with our transfer learning substantiated triple parameter training efficiency and better AF quality than our random parameter initialization. ConsTrain’s iterative parameter update is not convex due to our attached training hyper-parameters. Thus, we can conclude that providing well-defined scoring parameter values guided ConsTrain to better machine-learning solutions. In each test alignment dataset, ConsProb-trained showed higher SPSs than ConsProb-Turner, but the former was inferior to the latter regarding SCIs. From this observation, we can summarize that ConsTrain’s fully trained parameters outperformed our *ad hoc* parameters in simple matching quality while the latter was still more advantageous from a thermodynamic basis compared to the former. ConsProb-ensemble made flexible use of both ConsProb-trained and ConsProb-Turner to achieve ConsAlign’s robust AF quality. In other words, both thermodynamics-based and entirely statistical models compensated each other for predictive weak points. As a checkpoint of ConsTrain’s validity, we improved the sequence-based matching precision of our thermodynamics-based SAF model by transplanting transfer-learned sequence alignment parameters into ConsProb-Turner.

Recently, deep learning-based methods for folding single RNA sequences—such as SPOT-RNA (Singh et al. 2019), MXfold (Sato et al. 2021), and UFold (Fu et al. 2022)—emerged, replacing non-deep learning methods for folding single RNA sequences—such as CONTRAfold and TORNADO (Rivas et al. 2012). Expanding ConsTrain into deep learning for SAF has the potential to improve SAF’s quality by in-depth features unable to capture in any CLLMs. In another scenario, we have a chance to generalize the standard RNA base alphabet (A, C, G, and U) used in ConsTrain by including modified bases—such as I and Ψ (Harcourt et al. 2017). However, we need well-defined training datasets containing modified bases in this scenario because the base composition of training datasets directly affects ConsTrain’s trained scoring parameters via gradients. Constructing reliable large structural databases for modified bases can assist machine learning methods involving RNA secondary structures in data-driven avenues.

### 4.1 Notations and definitions

#### 4.1.1 Notations

*AF*: alignment and folding. *The BFGS algorithm*: the Broyden–Fletcher–Goldfarb–Shanno algorithm. *CLLM*: conditional log-linear model. *CRF*: conditional random field. *MCC*: Matthews correlation coefficient. *SAF*: simultaneous alignment and folding. *SCFG*: stochastic context-free grammar. *SCI*: structure conservation index. *SPS*: sum-of-pairs score.

### 4.2 Definitions

*CLLM*, *CRF*, and *SCFG*: They are probabilistic training frameworks for training data containing hidden sequential/structural states. CRFs are generalized variants of SCFGs, allowing each trained parameter not to be the (strict) form of a log probability. CLLMs generalize CRFs to loosen each learned parameter’s correspondence to a single transition/emission.

#### 4.2.1 Simultaneous structural alignment

It solves AF (i.e. structural alignment) of multiple RNA homologs in a unified scheme (e.g. a single dynamic programming scheme), not separately. One of the simultaneous alignment’s merits is that obtained alignments are globally optimum regarding any given scoring scheme compared to the case where we solve structural alignment separately.

#### 4.2.2 RNA loop structure

It is another view of RNA secondary structures based on structural features often found in them (e.g. hairpin loops and base pair stacking). Capturing RNA loop structures in scoring schemes involving secondary structures is significant including simultaneous alignment scoring, as Turner’s model (Turner and Mathews 2010), the CONTRAfold model (Do et al. 2006a), and ConsAlign do.

#### 4.2.3 Sparse dynamic programming

It computes and stores only preferable cells rather than all dynamic programming cells. We implemented our sparse dynamic programming based on Equation (1) by conforming to RAF’s implementation called the ‘alignment shell optimization’ (Do et al. 2008).

#### 4.2.4 γ-centroid estimator

It performs expected accuracy-first prediction given posterior probabilities. Since the space of potential solutions grows exponentially to input data’ length (e.g. RNAs’ length), maximizing likelihood (e.g. minimizing free energy) does not perform well for long input data in theory. The *γ*-centroid estimator maximizes potential solutions’ expected accuracy to deal with this ‘curse of dimensionality’ rather than maximizing their likelihood. As a side effect, *γ*-centroid estimators deliver possible solutions’ sparsity by leaving only posterior probabilities above a threshold (Hamada et al. 2009a; Sato et al. 2012), as DAFS and ConsAlign do.

## Supplementary Material

btad255_Supplementary_Data

## Data Availability

Our underlying data are available at our shown GitHub repositories.

## Appendix 1. Computing sparse posterior probabilities

### A.1 Computing sparse pair-matching probabilities

We distinguish the matching of base-pairing nucleotides (called pair-matching) and unpaired nucleotides (called loop-matching). For every two RNA sequences, ConsProb computes the posterior pair-matching probabilities upon sparse pairwise SAF using SAF scoring parameters θ (Tagashira and Asai 2022):

$$p_{\mathrm{ijkl}}(\theta )\overset{\text{def}}{=}\sum_{A\prime :A\prime \in A}p(A\prime ;\theta )I[(i,j,k,l)\in M^{P}(A\prime )].$$

Here, A is a sparse set of possible pairwise SAF candidates; $M^{P}(A\prime )$ is a set of pair-matching nucleotide quadruples in A′. ConsProb computes sparse posterior pair-matching probabilities $p_{\mathrm{ijkl}}(\theta )$ using its inside–outside algorithm:

$$p_{\mathrm{ijkl}}(\theta )\equiv \frac{\alpha_{\mathrm{ijkl}}(\theta )\beta_{\mathrm{ijkl}}(\theta )}{\alpha_{NM}(\theta )}.$$

Here, $\alpha_{\mathrm{ijkl}}(\theta )$ is the inside partition function corresponding to every four pair-matching nucleotides i,j,k,l (given SAF scoring parameters θ). (Each inside partition function covers possible stochastic states inside given indices, including these given indices.) Contrarily, $\beta_{\mathrm{ijkl}}(\theta )$ is the outside partition function corresponding to every four pair-matching nucleotides i,j,k,l. (Each outside partition function covers possible stochastic states outside given indices, excluding these given indices.) Finally, $\alpha_{NM}(\theta )$ is the forward partition function expressing (sub-)SAF from the nucleotides 1, 1 to the nucleotides *N*, *M*. (Each forward partition function covers possible stochastic states ending at given indices, including these given indices.).

### A.2 Computing sparse loop-matching probabilities

Following ConsProb’s computation of sparse posterior pair-matching probabilities $p_{\mathrm{ijkl}}(\theta )$, we add the computation of sparse posterior loop-matching probabilities to ConsProb:

$$p_{uv}(\theta )\overset{\text{def}}{=}\sum_{A\prime :A\prime \in A}p(A\prime ;\theta )I[(u,v)\in M^{L}(A\prime )].$$

Here, $M^{L}(A\prime )$ is a set of loop-matching nucleotide pairs in A′. More specifically, we calculate the following stochastic variable as any sparse loop-matching probability

$$p_{uv}(\theta )\equiv \frac{1}{\alpha_{NM}(\theta )}\sum_{\mathrm{ijkl}s}\beta_{\mathrm{ijkl}}(\theta )\alpha_{uv}^{s}(\theta ;i,j,k,l)\beta_{uv}^{s}(\theta ;i,j,k,l).$$

Here, $\alpha_{uv}^{s}(\theta ;i,j,k,l)$ is the forward partition function expressing sub-SAF given a structural context s (e.g., a hairpin loop and a bulge loop) from nucleotides *i*, *k* to nucleotides *u*, *v*, where *i*, *k* pair-match nucleotides j,l:u<j,v<l; $\beta_{uv}^{s}(\theta ;i,j,k,l)$ is the backward partition function expressing sub-SAF given s from *u*, *v* to *j*, *l*, where *j*, *l* pair-match nucleotides i,k:i<u,k<v. (Each backward partition function covers possible stochastic states beginning at given indices, excluding these given indices.). To cover all possible unpaired nucleotides upon partition functions $\alpha_{uv}^{s}(\theta ;i,j,k,l),\beta_{uv}^{s}(\theta ;i,j,k,l)$, we add two base-pairing sentinel nucleotides 0,N+1 or 0,M+1 to both ends of each RNA sequence.

### A.3 Converting probabilities for AF prediction

We define $p_{\mathrm{ijkl}}(\theta ;x,y)$ as the explicit form of each pair-matching probability $p_{\mathrm{ijkl}}(\theta )$ regarding every two RNA homologs *x*, *y* and convert $p_{\mathrm{ijkl}}(\theta ;x,y)$ into average probabilistic consistency (Do et al. 2005; Tagashira and Asai 2022)

$$p_{ij}^{P}(\theta )\overset{\text{def}}{=}p_{ij}^{P}(\theta ;x)\overset{\text{def}}{=}\frac{1}{|R|-1}\sum_{\mathrm{ykl}:y\in R,y\neq x}p_{\mathrm{ijkl}}(\theta ;x,y).$$

Here, *R* is a set of all RNA homologs to be aligned. Average sparse probabilistic consistency $p_{ij}^{P}(\theta ;x)$ expresses the base-pairing capability of every two nucleotides *i*, *j* in each RNA homolog *x*, considering all the other RNA homologs *y*. We also obtain sparse posterior matching probability by combining posterior probabilities $p_{ik}(\theta ),p_{\mathrm{ijkl}}(\theta )$:

$$p_{ik}^{M}(\theta )\overset{\text{def}}{=}p_{ik}(\theta )+\sum_{jl:i<j,k<l}p_{\mathrm{ijkl}}(\theta )+\sum_{jl:j<i,l<k}p_{\mathrm{jilk}}(\theta ).$$

## References

[btad255-B1] Aghaeepour N , Hoos HH. Ensemble-based prediction of RNA secondary structures. BMC Bioinformatics 2013;14:139. 10.1186/1471-2105-14-139. 23617269 PMC3750279

[btad255-B2] Andronescu M , Condon A, Hoos HH et al Efficient parameter estimation for RNA secondary structure prediction. Bioinformatics 2007;23:i19–28. 10.1093/bioinformatics/btm223. 17646296

[btad255-B3] Bernhart SH , Hofacker IL, Will S et al RNAalifold: improved consensus structure prediction for RNA alignments. BMC Bioinformatics 2008;9:474. 10.1186/1471-2105-9-474. 19014431 PMC2621365

[btad255-B4] Cleveland WS. LOWESS: a program for smoothing scatterplots by robust locally weighted regression. Am Stat 1981;35:54. 10.2307/2683591.

[btad255-B5] Ding Y , Chi YC, Lawrence CE. RNA secondary structure prediction by centroids in a Boltzmann weighted ensemble. RNA 2005;11:1157–66. 10.1261/rna.2500605. 16043502 PMC1370799

[btad255-B6] Do CB , Foo C, Batzoglou S. A max-margin model for efficient simultaneous alignment and folding of RNA sequences. Bioinformatics 2008;24:i68–76. 10.1093/bioinformatics/btn177. 18586747 PMC2718655

[btad255-B7] Do CB , Gross SS, Batzoglou S. CONTRAlign: discriminative training for protein sequence alignment. In: *Proceedings of the Tenth Annual International Conference on Computational Molecular Biology (RECOMB 2006)*, Venice, Italy. Cham: Springer, 2006b, 160–74.

[btad255-B8] Do CB , Mahabhashyam MSP, Brudno M et al ProbCons: probabilistic consistency-based multiple sequence alignment. Genome Res 2005;15:330–40. 10.1101/gr.2821705. 15687296 PMC546535

[btad255-B9] Do CB , Woods DA, Batzoglou S. CONTRAfold: RNA secondary structure prediction without physics-based models. Bioinformatics2006a;22:e90–8. 10.1093/bioinformatics/btl246. 16873527

[btad255-B10] Dowell RD , Eddy SR. Efficient pairwise RNA structure prediction and alignment using sequence alignment constraints. BMC Bioinformatics 2006;7:400. 10.1186/1471-2105-7-400. 16952317 PMC1579236

[btad255-B11] Feng D , Doolittle RF. Progressive sequence alignment as a prerequisitetto correct phylogenetic trees. J Mol Evol 1987;25:351–60. 10.1007/BF02603120. 3118049

[btad255-B12] Fletcher R. Practical Methods of Optimization. New York, NY: John Wiley & Sons, Ltd, 1987.

[btad255-B13] Foo CS , Do CB, Ng AY. A majorization-minimization algorithm for (multiple) hyperparameter learning. In: *Proceedings of the 26th International Conference on Machine Learning 2009*, Quebec, Canada, Association for Computing Machinery, 2009, 321–8.

[btad255-B14] Fu L , Cao Y, Wu J et al UFold: fast and accurate RNA secondary structure prediction with deep learning. Nucleic Acids Res 2022;50:e14. 10.1093/nar/gkab1074. 34792173 PMC8860580

[btad255-B15] Fukunaga T , Ozaki H, Terai G et al CapR: Revealing structural specificities of RNA-binding protein target recognition using CLIP-seq data. Genome Biol 2014;15:R16. 10.1186/gb-2014-15-1-r16. 24447569 PMC4053987

[btad255-B16] Gardner PP , Wilm A, Washietl S. A benchmark of multiple sequence alignment programs upon structural RNAs. Nucleic Acids Res 2005;33:2433–9. 10.1093/nar/gki541. 15860779 PMC1087786

[btad255-B17] Hamada M , Kiryu H, Sato K et al Prediction of RNA secondary structure using generalized centroid estimators. Bioinformatics2009a;25:465–73. 10.1093/bioinformatics/btn601. 19095700

[btad255-B18] Hamada M , Sato K, Asai K. Improving the accuracy of predicting secondary structure for aligned RNA sequences. Nucleic Acids Res 2011;39:393–402. 10.1093/nar/gkq792. 20843778 PMC3025558

[btad255-B19] Hamada M , Sato K, Asai K. Prediction of RNA secondary structure by maximizing pseudo-expected accuracy. BMC Bioinformatics 2010;11:586. 10.1186/1471-2105-11-586. 21118522 PMC3003279

[btad255-B20] Hamada M , Sato K, Kiryu H et al CentroidAlign: fast and accurate aligner for structured RNAs by maximizing expected sum-of-pairs score. Bioinformatics2009c;25:3236–43. 10.1093/bioinformatics/btp580. 19808876

[btad255-B21] Hamada M , Sato K, Kiryu H et al Predictions of RNA secondary structure by combining homologous sequence information. Bioinformatics2009b;25:i330–8. 10.1093/bioinformatics/btp228. 19478007 PMC2687982

[btad255-B22] Harcourt EM , Kietrys AM, Kool ET. Chemical and structural effects of base modifications in messenger RNA. Nature 2017;541:339–46. 10.1038/nature21351. 28102265 PMC5498787

[btad255-B23] Harmanci AO , Sharma G, Mathews DH. TurboFold: iterative probabilistic estimation of secondary structures for multiple RNA sequences. BMC Bioinformatics 2011;12:108. 10.1186/1471-2105-12-108. 21507242 PMC3120699

[btad255-B24] Hofacker IL , Bernhart SHF, Stadler PF. Alignment of RNA base pairing probability matrices. Bioinformatics 2004;20:2222–7. 10.1093/bioinformatics/bth229. 15073017

[btad255-B25] Jabbari H , Wark I, Montemagno C et al Knotty: efficient and accurate prediction of complex RNA pseudoknot structures. Bioinformatics 2018;34:3849–56. 10.1093/bioinformatics/bty420. 29868872

[btad255-B26] Kalvari I , Argasinska J, Quinones-Olvera N et al Rfam 13.0: shifting to a genome-centric resource for non-coding RNA families. Nucleic Acids Res 2018;46:D335–42. 10.1093/nar/gkx1038. 29112718 PMC5753348

[btad255-B27] Kiryu H , Tabei Y, Kin T et al Murlet: a practical multiple alignment tool for structural RNA sequences. Bioinformatics 2007;23:1588–98. 10.1093/bioinformatics/btm146. 17459961

[btad255-B28] Knudsen B , Hein J. Pfold: RNA secondary structure prediction using stochastic context-free grammars. Nucleic Acids Res 2003;31:3423–8. 10.1093/nar/gkg614. 12824339 PMC169020

[btad255-B29] Lafferty J , McCallum A, Pereira F. Conditional random fields: probabilistic models for segmenting and labeling sequence data. In: *Proceedings of the 18th International Conference on Machine Learning 2001*, MA, USA. Morgan Kaufmann Publishers Inc., 2001, 282–289.

[btad255-B30] Lange K , Hunter DR, Yang I. Optimization transfer using surrogate objective functions. J Comput Graph Stat 2000;9:1–20. 10.2307/1390605.

[btad255-B31] Li S , Zhang H et al LinearTurboFold: linear-time global prediction of conserved structures for RNA homologs with applications to SARS-CoV-2. Proc Natl Acad Sci USA 2021;118:e2116269118. 10.1073/pnas.211626911. 34887342 PMC8719904

[btad255-B32] Nawrocki EP , Eddy SR. Infernal 1.1: 100-fold faster RNA homology searches. Bioinformatics 2013;29:2933–5. 10.1093/bioinformatics/btt509. 24008419 PMC3810854

[btad255-B34] Rivas E , Lang R, Eddy SR. A range of complex probabilistic models for RNA secondary structure prediction that includes the nearest-neighbor model and more. RNA 2012;18:193–212. 10.1261/rna.030049.111. 22194308 PMC3264907

[btad255-B35] Sankoff D. Simultaneous solution of the RNA folding, alignment and protosequence problems. SIAM J Appl Math 1985;45:810–25. 10.1137/0145048.

[btad255-B36] Sato K , Akiyama M, Sakakibara Y. RNA secondary structure prediction using deep learning with thermodynamic integration. Nat Commun 2021;12:9. 10.1038/s41467-021-21194-4. 33574226 PMC7878809

[btad255-B37] Sato K , Kato Y. Prediction of RNA secondary structure including pseudoknots for long sequences. Brief Bioinform 2022;23:1–9. 10.1093/bib/bbab395. PMC8769711 34601552

[btad255-B38] Sato K , Kato Y, Akutsu T et al DAFS: simultaneous aligning and folding of RNA sequences via dual decomposition. Bioinformatics 2012;28:3218–24. 10.1093/bioinformatics/bts612. 23060618

[btad255-B39] Sato K , Kato Y, Hamada M et al IPknot: fast and accurate prediction of RNA secondary structures with pseudoknots using integer programming. Bioinformatics 2011;27:i85–93. 10.1093/bioinformatics/btr215. 21685106 PMC3117384

[btad255-B40] Sato K , Sakakibara Y. RNA secondary structural alignment with conditional random fields. Bioinformatics 2005;21:ii237–42. 10.1093/bioinformatics/bti1139. 16204111

[btad255-B41] Seemann SE , Gorodkin J, Backofen R. Unifying evolutionary and thermodynamic information for RNA folding of multiple alignments. Nucleic Acids Res 2008;36:6355–62. 10.1093/nar/gkn544. 18836192 PMC2582601

[btad255-B42] Singh J , Hanson J, Paliwal K et al RNA secondary structure prediction using an ensemble of two-dimensional deep neural networks and transfer learning. Nat Commun 2019;10:13. 10.1038/s41467-019-13395-9. 31776342 PMC6881452

[btad255-B43] Sneath PH , Sokal RR. Numerical taxonomy. Nature 1962;193:855–60. 10.1038/193855a0. 13914561

[btad255-B44] Tagashira M , Asai K. ConsAlifold: considering RNA structural alignments improves prediction accuracy of RNA consensus secondary structures. Bioinformatics 2022;38:710–9. 10.1093/bioinformatics/btab738. 34694364

[btad255-B45] Tan Z , Fu Y, Sharma G et al TurboFold II: RNA structural alignment and secondary structure prediction informed by multiple homologs. Nucleic Acids Res 2017;45:11570–81. 10.1093/nar/gkx815. 29036420 PMC5714223

[btad255-B46] Thompson JD , Plewniak F, Poch O. A comprehensive comparison of multiple sequence alignment programs. Nucleic Acids Res 1999;27:2682–90. 10.1093/nar/27.13.2682. 10373585 PMC148477

[btad255-B47] Tsochantaridis I , Joachims T, Hofmann T et al Large margin methods for structured and interdependent output variables. J Mach Learn Res 2005;6:1453–84.

[btad255-B48] Turner DH , Mathews DH. NNDB: the nearest neighbor parameter database for predicting stability of nucleic acid secondary structure. Nucleic Acids Res 2010;38:D280–2. 10.1093/nar/gkp892. 19880381 PMC2808915

[btad255-B49] Washietl S , Hofacker IL, Stadler PF. Fast and reliable prediction of noncoding RNAs. Proc Natl Acad Sci USA 2005;102:2454–9. 10.1073/pnas.0409169102. 15665081 PMC548974

[btad255-B50] Will S , Joshi T, Hofacker IL et al LocARNA-P: accurate boundary prediction and improved detection of structural RNAs. RNA 2012;18:900–14. 10.1261/rna.029041.111. 22450757 PMC3334699

[btad255-B51] Will S , Otto C, Miladi M et al SPARSE: quadratic time simultaneous alignment and folding of RNAs without sequence-based heuristics. Bioinformatics 2015;31:2489–96. 10.1093/bioinformatics/btv185. 25838465 PMC4514930

[btad255-B52] Will S , Reiche K, Hofacker IL et al Inferring noncoding rna families and classes by means of genome-scale structure-based clustering. PLoS Comput Biol 2007;3:680–91. 10.1371/journal.pcbi.0030065. PMC1851984 17432929

[btad255-B53] Zakov S , Goldberg Y, Elhadad M et al Rich parameterization improves RNA structure prediction. J Comput Biol 2011;18:1525–42. 10.1089/cmb.2011.0184. 22035327

