# Supplementary material for: ConsAlign: simultaneous RNA structural aligner based on rich transfer learning and thermodynamic ensemble model of alignment scoring
Source: Bioinformatics. 2023 Apr 19;39(5):btad255. doi: 10.1093/bioinformatics/btad255 (PMC10172041; doi:10.1093/bioinformatics/btad255)
Supplement: btad255_Supplementary_Data [file btad255_supplementary_data.pdf]

S1 Computing expected parameter occurrences

We can calculate approximate expected parameter occurrences $\mathbb{E}_d^{\mathrm{S}}[\boldsymbol{\phi}(\cdot);\boldsymbol{\theta}]$ based on ConsProb's sparse posterior probabilities; for example, $\mathbb{E}_d^{\mathrm{S}}[\boldsymbol{\phi}(\cdot);\boldsymbol{\theta}]$'s element corresponding to CONTRAlign's matching emission parameter $\theta_{\mathtt{AA}}^{\mathrm{match}}$ is

$$\sum_{uv} p_{uv}(\boldsymbol{\theta}; d) I\{[t(u), t(v)] = (\mathtt{A}, \mathtt{A})\}.$$

Here, $p_{uv}(\boldsymbol{\theta}; d)$ is the explicit form of each sparse loop-matching probability $p_{uv}(\boldsymbol{\theta})$ regarding each d-th RNA sequence pair. Also, $t(u)$ returns the type of any nucleotide (position) u and is $t(u) \in \{\mathtt{A}, \mathtt{C}, \mathtt{G}, \mathtt{U}\}$. As another example, we can compute counts $\mathbb{E}_d^{\mathrm{S}}[\boldsymbol{\phi}(\cdot);\boldsymbol{\theta}]$'s element corresponding to CONTRAfold's hairpin loop length $\theta_{30}^{\mathrm{hairpin\ length}}$ as follows:

$$\sum_{ijkl} p_{ijkl}^{\mathrm{hairpin\ loop}}(\boldsymbol{\theta}; d) I(|j - i - 1| = 30) + \sum_{ijkl} p_{ijkl}^{\mathrm{hairpin\ loop}}(\boldsymbol{\theta}; d) I(|l - k - 1| = 30).$$

Here, $p_{ijkl}^{\mathrm{hairpin\ loop}}(\boldsymbol{\theta}; d)$ is the explicit form of each posterior pair-matching probability $p_{ijkl}(\boldsymbol{\theta})$ regarding each d-th RNA sequence pair and assumes that every two base pairings $(i, j), (k, l)$ enclose two hairpin loops. [ConsProb can calculate $p_{ijkl}^{\mathrm{hairpin\ loop}}(\boldsymbol{\theta}; d)$ during the computation of $p_{ijkl}(\boldsymbol{\theta})$.]

S2 Settings of trained SAF scoring model

S2.1 Decomposing ConsTrain's SAF scoring model

CONTRAfold's parameters $\boldsymbol{\theta}_{\mathrm{fold}}$ score any secondary structure $\boldsymbol{S}$ as follows (Do *et al.*, 2006a):

$$s^{\mathrm{fold}}(\boldsymbol{S}; \boldsymbol{\theta}_{\mathrm{fold}}) \stackrel{\mathrm{def}}{=} \boldsymbol{\theta}_{\mathrm{fold}}^{\mathrm{T}} \cdot \boldsymbol{\phi}^{\mathrm{fold}}(\boldsymbol{S}).$$

Here, a function $\boldsymbol{\phi}^{\mathrm{fold}}(\boldsymbol{S})$ maps any secondary structure $\boldsymbol{S}$ to a vector that counts the occurrence of each f-th scoring parameter $\theta_f^{\mathrm{fold}} : (\theta_f^{\mathrm{fold}}) \equiv \boldsymbol{\theta}^{\mathrm{fold}}$ in $\boldsymbol{S}$. Likewise, CONTRAlign's parameters $\boldsymbol{\theta}_{\mathrm{align}}$ score any pairwise sequence alignment $\boldsymbol{B}$ in the following form (Do *et al.*, 2006b):

$$s^{\mathrm{align}}(\boldsymbol{B}; \boldsymbol{\theta}_{\mathrm{align}}) \stackrel{\mathrm{def}}{=} \boldsymbol{\theta}_{\mathrm{align}}^{\mathrm{T}} \cdot \boldsymbol{\phi}^{\mathrm{align}}(\boldsymbol{B}).$$

Here, a function $\boldsymbol{\phi}^{\mathrm{align}}(\boldsymbol{B})$ maps any pairwise sequence alignment $\boldsymbol{B}$ to the vector that counts the occurrence of each f-th scoring parameter $\theta_f^{\mathrm{align}} : (\theta_f^{\mathrm{align}}) \equiv \boldsymbol{\theta}^{\mathrm{align}}$ in $\boldsymbol{B}$. Any pairwise SAF candidate $\mathbb{A}$ of every two RNA sequences is composed of (1) some secondary structure $\boldsymbol{S}$ of one of them, (2) some secondary structure $\boldsymbol{S}'$ of the other of them, and (3) some pairwise sequence alignment $\boldsymbol{B}$ of them:

$$\mathbb{A} \stackrel{\mathrm{def}}{=} (\boldsymbol{S}, \boldsymbol{S}', \boldsymbol{B}).$$

We specify the form of our SAF scoring parameters $\boldsymbol{\theta}$ by concatenating CONTRAfold and CONTRAlign's parameters $\boldsymbol{\theta}_{\mathrm{fold}}, \boldsymbol{\theta}_{\mathrm{align}}$:

$$\boldsymbol{\theta} \stackrel{\mathrm{def}}{=} \boldsymbol{\theta}_{\mathrm{fold}} \oplus \boldsymbol{\theta}_{\mathrm{align}}.$$

Finally, we can rewrite our SAF scoring $s(\mathbb{A}; \boldsymbol{\theta})$ using CONTRAfold and CONTRAlign's parameters $\boldsymbol{\theta}_{\mathrm{fold}}, \boldsymbol{\theta}_{\mathrm{align}}$:

$$\begin{aligned} s(\mathbb{A}; \boldsymbol{\theta}) &\equiv \boldsymbol{\theta}_{\mathrm{fold}}^{\mathrm{T}} \cdot [\boldsymbol{\phi}^{\mathrm{fold}}(\boldsymbol{S}) + \boldsymbol{\phi}^{\mathrm{fold}}(\boldsymbol{S}')] + \boldsymbol{\theta}_{\mathrm{align}}^{\mathrm{T}} \cdot \boldsymbol{\phi}^{\mathrm{align}}(\boldsymbol{B}) \\ &\equiv s^{\mathrm{fold}}(\boldsymbol{S}; \boldsymbol{\theta}_{\mathrm{fold}}) + s^{\mathrm{fold}}(\boldsymbol{S}'; \boldsymbol{\theta}_{\mathrm{fold}}) + s^{\mathrm{align}}(\boldsymbol{B}; \boldsymbol{\theta}_{\mathrm{align}}). \end{aligned}$$

S2.2 CONTRAfold model

CONTRAfold's parameters $\boldsymbol{\theta}_{\mathrm{fold}}$ decompose any secondary structure $\boldsymbol{S}$ into its set of *loop*s $\mathcal{L}(\boldsymbol{S})$ to score $\boldsymbol{S}$. CONTRAfold's parameters $\boldsymbol{\theta}_{\mathrm{fold}}$ assign a different score to each loop $L : L \in \mathcal{L}(\boldsymbol{S})$ based on the class c_L of L [one of the external loops, hairpin loops, stackings, bulge loops, interior loops, and multi-loops (Fig. S1)]:

$$s(\boldsymbol{S}; \boldsymbol{\theta}_{\mathrm{fold}}) \stackrel{\mathrm{def}}{=} \sum_{L : L \in \mathcal{L}(\boldsymbol{S})} s^{c_L}(L; \boldsymbol{\theta}_{\mathrm{fold}}).$$

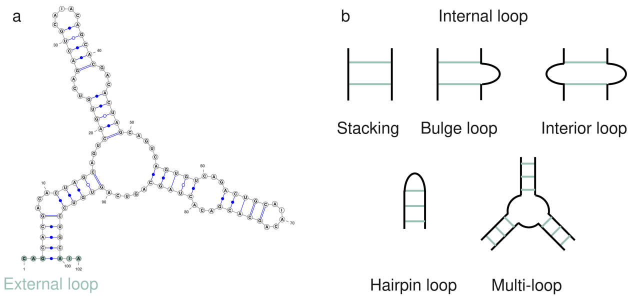

Fig. S1. (a) Each loop in any RNA secondary structure is either external or internal. The external loop of any RNA secondary structure covers the outmost nucleotides not enclosed by any base pairing. Internal loops cover all the nucleotides not covered by external loops. (b) Internal loops are classified into hairpin loops, stackings, bulge loops, interior loops, and multi-loops.

We characterize each loop scoring function $s^{c_L}(L; \boldsymbol{\theta}_{\mathrm{fold}})$ with loop-specific parameters in CONTRAfold's parameters $\boldsymbol{\theta}_{\mathrm{fold}}$ (Table S1). For example, we can compute a hairpin loop L with its length $|L|$ (i.e., the number of unpaired nucleotides) using the hairpin loop scoring function $s^{\mathrm{hairpin\ loop}}(L; \boldsymbol{\theta}_{\mathrm{fold}})$ as follows:

$$s^{\mathrm{hairpin\ loop}}(L; \boldsymbol{\theta}_{\mathrm{fold}}) \stackrel{\mathrm{def}}{=} \sum_{x : x \in \{0, \ldots, |L|\}} \theta_x^{\mathrm{hairpin\ length}} + \theta_{p_1}^{\mathrm{helix\ end}} + \theta_{p_1 p_2}^{\mathrm{terminal\ mismatch}}.$$

Here, p_1 is the base-pairing enclosing any hairpin loop L, and p_2 is the unpaired nucleotide pair neighboring p_1 (called terminal mismatches). Moreover, $\theta_x^{\mathrm{hairpin\ length}}$ scores hairpin loops with the lengths of at least x; $\theta_{p_1}^{\mathrm{helix\ end}}$ scores the helix end formed by any base-pairing p_1; $\theta_{p_1 p_2}^{\mathrm{terminal\ mismatch}}$ scores the terminal mismatch end formed by every two nucleotide pairs p_1, p_2.

S2.3 CONTRAlign model

Based on the pair-conditional random field shown in Fig. S2, CONTRAlign's parameters $\boldsymbol{\theta}_{\mathrm{align}}$ assign different scores to the emissions and the transitions involving nucleotide matches and nucleotide indels (Table S1). For example, we score the sequence alignment below

```
ACCGU--GU

AC--UUUGU
```

by summing the CONTRAlign parameters appearing in it:

$$\begin{aligned} &\theta^{\mathrm{init\ match}} + \theta^{\mathrm{indel} \to \mathrm{match}} \\ &+ 2(\theta^{\mathrm{match} \to \mathrm{match}} + \theta^{\mathrm{match} \to \mathrm{indel}} + \theta^{\mathrm{indel\ extend}}) \\ &+ \theta_{\mathtt{AA}}^{\mathrm{match}} + \theta_{\mathtt{CC}}^{\mathrm{match}} + \theta_{\mathtt{GG}}^{\mathrm{match}} + 2\theta_{\mathtt{UU}}^{\mathrm{match}} \\ &+ \theta_{\mathtt{C}}^{\mathrm{indel}} + \theta_{\mathtt{G}}^{\mathrm{indel}} + 2\theta_{\mathtt{U}}^{\mathrm{indel}}. \end{aligned}$$

Table S1. Specified types of our SAF scoring parameters.

SAF scoring parameter group	Group size	Source
Hairpin loop length	31	CONTRAfold
Bulge loop length	30	
Interior loop length	29	
Interior loop length symmetric	15	
Interior loop length asymmetric	28	
Stacking	21	
Terminal mismatch	96	
		⋮
Dangling	48	
Helix end	6	
Base-pairing	3	
Interior loop length explicit	10	
Bulge loop length 0×1	4	
Interior loop length 1×1	10	
Multi-loop length	3	
External-loop length	2	CONTRAfold
Match transition	3	CONTRAlign
		⋮
Indel transition	2	
Indel emission	4	
Match emission	10	CONTRAlign
Total (from 19 groups)	355	

Our SAF pair-CLLM combines the scoring parameter forms of the CONTRAfold model and the CONTRAlign model. Our pair-CLLM presumes the standard RNA nucleotide alphabet A, C, G, U and the canonical RNA base-pairing alphabet AU, CG, GU, UA, GC, UG.

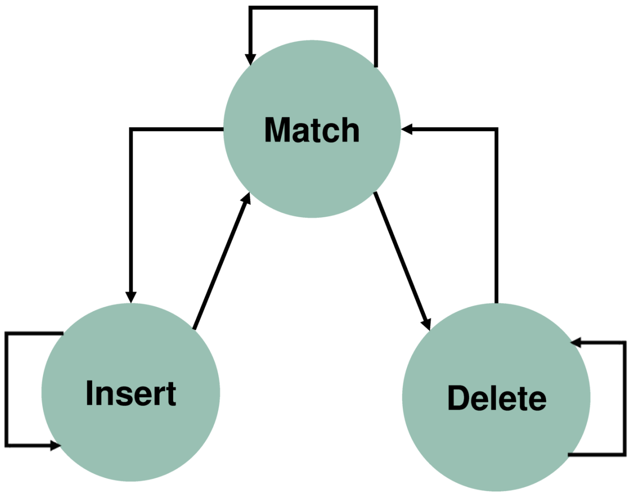

Fig. S2. Pair-conditional random field casts CONTRAlign's scoring parameters against any pairwise sequence alignment.

S3 Conventional AF tools' features

(1) RAF (a) is a SAF tool taking both CONTRAfold and CONTRAlign's posterior probabilities and (b) trains the weight parameters of these posterior probabilities in max-margin optimization (Do *et al.*, 2008). (2, 3) LocARNA and SPARSE are different implementations of Sankoff's algorithm utilizing SAF sparsity (Will *et al.*, 2007, 2015). SPARSE is a variant of LocARNA and exploits only structure-based constraints (Will *et al.*, 2015), whereas LocARNA exploits matching-based and structure-based constraints (Will *et al.*, 2007). (4) DAFS is a SAF tool and realizes reasonable computational complexities by applying dual decomposition to integer programming (Sato *et al.*, 2012). (5) LinearTurboFold is an application of LinearPartition (Zhang *et al.*, 2020) to TurboFold, an iterative AF tool (Tan *et al.*, 2017). LinearTurboFold realizes its quick predictive iteration using both LinearPartition and beam search-based sequence alignment (Li *et al.*, 2021).

S4 Algorithms

Algorithm S1 Algorithm that optimizes hyper-parameters $\gamma^{\mathrm{M}}, \gamma^{\mathrm{P}}$ together with predicted SAF.

function alignWrapper(RNA homolog sequences to be aligned, SAF scoring parameters $\boldsymbol{\theta}$)
  for $i \in \{i^{\min}, \ldots, i^{\max}\}, j \in \{j^{\min}, \ldots, j^{\max}\}$ **do**
    $\gamma^{\mathrm{M}} \leftarrow 2^i + 1, \gamma^{\mathrm{P}} \leftarrow 2^j + 1$
    Predict a SAF candidate $\mathbb{A}$ using the progressive SAF scheme, given $\gamma^{\mathrm{M}}, \gamma^{\mathrm{P}}$
    Compute the expected sum-of-pairs score $\mathbb{E}[\sigma(\mathbb{A}, \cdot); \boldsymbol{\theta}]$ of $\mathbb{A}$
    $\sigma^{\mathrm{new}} \leftarrow \mathbb{E}[\sigma(\mathbb{A}, \cdot); \boldsymbol{\theta}]$
    if $\sigma^{\mathrm{new}} > \sigma^{\mathrm{curr}}$ or σ^{curr} is unset **then**
      $\mathbb{A}^* \leftarrow \mathbb{A}, \gamma^{\mathrm{M},*} \leftarrow \gamma^{\mathrm{M}}, \gamma^{\mathrm{P},*} \leftarrow \gamma^{\mathrm{P}}, \sigma^{\mathrm{curr}} \leftarrow \sigma^{\mathrm{new}}$
  return $(\mathbb{A}^*, \gamma^{\mathrm{M},*}, \gamma^{\mathrm{P},*})$

Algorithm S2 BFGS algorithm that minimizes an approximated convex pair-CLLM cost $c^{\mathrm{S}}(D; \boldsymbol{\theta})$.

function bfgsAlgo(a training dataset D)
  Initialize SAF scoring parameters $\boldsymbol{\theta}^*$
  for $d \in \{1, \ldots, |D|\}$ **do**
    Get parameter counts $\boldsymbol{\phi}(\mathbb{A}_d)$ by parsing each training AF example $\mathbb{A}_d$
  while $\boldsymbol{\theta}^*$ does not converge **do**
    for $d \in \{1, \ldots, |D|\}$ **do**
      Compute expected counts $\mathbb{E}^{\mathrm{S}}_d[\boldsymbol{\phi}(\cdot); \boldsymbol{\theta}^*]$ using our inside-outside algorithm
    $\nabla c^{\mathrm{S}}(D; \boldsymbol{\theta}^*) \leftarrow \sum_{d:d \in \{1,\ldots,|D|\}} \{\mathbb{E}^{\mathrm{S}}_d[\boldsymbol{\phi}(\cdot); \boldsymbol{\theta}^*] - \boldsymbol{\phi}(\mathbb{A}_d)\}$
    /* Apply the parameter update scheme of the BFGS algorithm */
    $\boldsymbol{\theta}^* \leftarrow \mathrm{argmin}^{\mathrm{BFGS}}[c^{\mathrm{S}}(D; \boldsymbol{\theta}^*) | \nabla c^{\mathrm{S}}(D; \boldsymbol{\theta}^*)]$
  return $\boldsymbol{\theta}^*$

Algorithm S3 Grouped majorization-minimization algorithm that minimizes an L2-regularized non-convex cost $c^{\mathrm{R}}(D; \boldsymbol{\theta}, \alpha, \beta)$.

function groupedMmAlgo(a training dataset D, the two gamma distribution parameters α, β)
  Initialize SAF scoring parameters $\boldsymbol{\theta}^*$
  $\boldsymbol{\lambda} \leftarrow \mathbf{1}$ /* $\mathbf{1}$ is a vector composed only of ones */
  while $\boldsymbol{\theta}^*$ does not converge **do**
    $\nabla c^{\mathrm{S}}(D; \boldsymbol{\theta}^*) \leftarrow \sum_{d:d \in \{1,\ldots,|D|\}} \{\mathbb{E}^{\mathrm{S}}_d[\boldsymbol{\phi}(\cdot); \boldsymbol{\theta}^*] - \boldsymbol{\phi}(\mathbb{A}_d)\}$
    $\nabla c^{\mathrm{R}}(D; \boldsymbol{\theta}^*, \alpha, \beta) \leftarrow \nabla c^{\mathrm{S}}(D; \boldsymbol{\theta}^*) + \boldsymbol{\lambda}^{\mathrm{T}} \cdot \boldsymbol{\theta}^*$
    $\boldsymbol{\theta}^* \leftarrow \mathrm{argmin}^{\mathrm{BFGS}}[c^{\mathrm{R}}(D; \boldsymbol{\theta}^*, \alpha, \beta) | \nabla c^{\mathrm{R}}(D; \boldsymbol{\theta}^*, \alpha, \beta)]$
    /* $(\theta^*_f) \stackrel{\mathrm{def}}{=} \boldsymbol{\theta}^*$ */
    $\boldsymbol{\lambda} \leftarrow (\frac{\frac{|G(f)|}{2} + \alpha}{\beta + \frac{1}{2} \sum_{f':f' \in \{1,\ldots,F\} | G(f') = G(f)} (\theta^*_{f'})^2})$
  return $\boldsymbol{\theta}^*$

S5 Figures

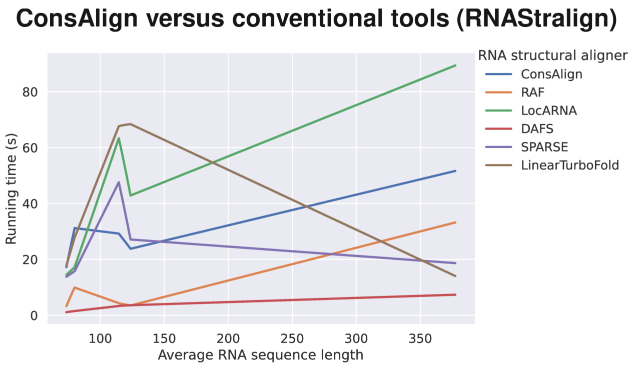

Fig. S3. The same as Fig. 6a, except that the dataset "RNAStralign processed" was used instead of the dataset "Rfam test." Lowess curves regarding the numbers of RNA sequences were unavailable since most test AF examples were formed by sets of 20 RNA sequences.

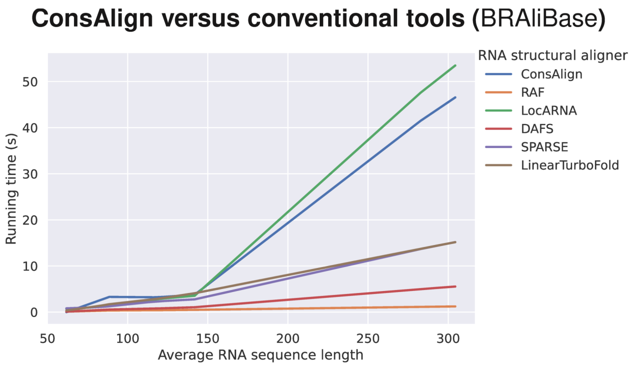

Fig. S4. The same as Fig. 6a, except that BRAliBase's dataset 1 was used instead of the dataset "Rfam test." Lowess curves regarding the numbers of RNA sequences were unavailable since each test alignment was formed by five RNA sequences.

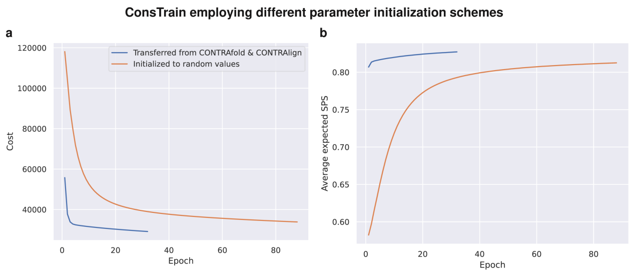

Fig. S5. (a) Decrement of our L2-regularized non-convex cost and (b) increment of average expected SPS in different parameter initialization settings. As the first choice, SAF scoring parameters were transfer-learned. As the second choice, SAF scoring parameters were initialized by random values. The dataset "Rfam train" was used.

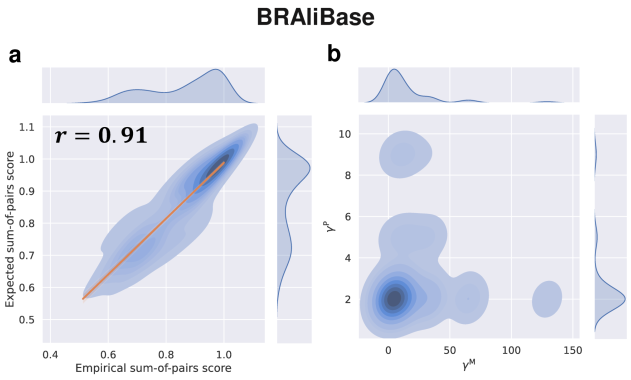

Fig. S6. The same as (a) Fig. 7 and (b) Fig. 8, except that BRAliBase's dataset 1 was used instead of the datasets "Rfam test" and "RNAStralign processed."

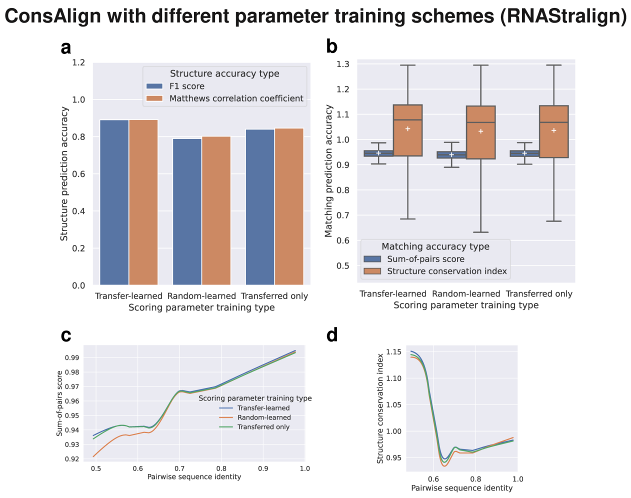

Fig. S7. The same as Fig. 9, except that the dataset "RNAStralign processed" was used instead of the dataset "Rfam test."

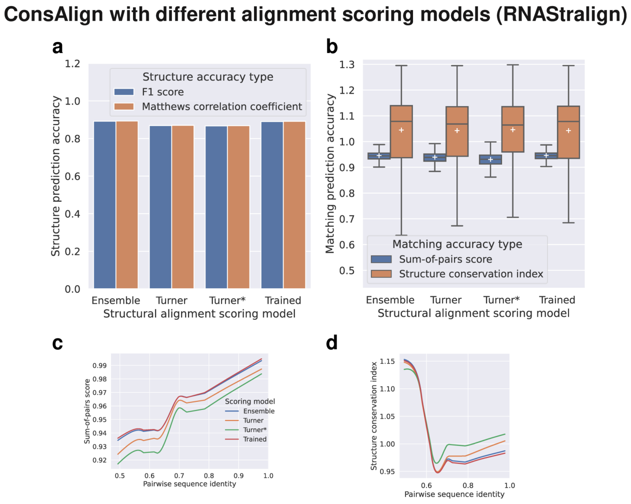

Fig. S8. The same as Fig. 10, except that the dataset "RNAStralign processed" was used instead of the dataset "Rfam test."

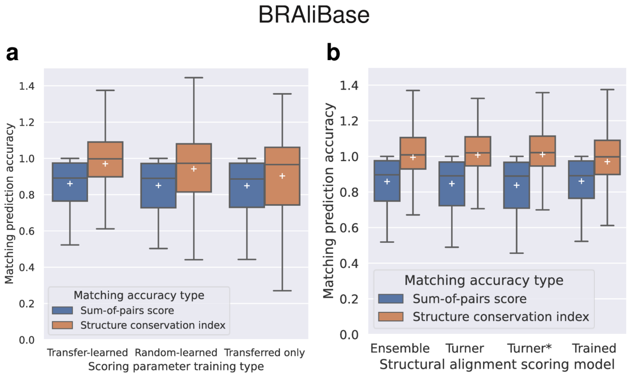

Fig. S9. The same as (a) Fig. 9b and (b) Fig. 10b, except that BRAliBase's dataset 1 was used instead of the dataset "Rfam test."

S6 Tables

Table S2. ConsAlign's two-sided, paired t-tests to conventional AF tools using the dataset "Rfam test."

Conventional tool	SPS-based p-value	SCI-based p-value
RAF	0.0008 (***)	0.0018 (***)
LocARNA	7.2×10^{-24} (***)	0.00012 (***)
DAFS	0.19	0.00012 (***)
SPARSE	1.4×10^{-104} (***)	0.74
LinearTurboFold	8.8×10^{-10} (***)	2.3×10^{-29} (***)

"***" indicates that a corresponding p-value is less than the significance level $\alpha \leftarrow 0.001$. `stats.ttest_rel()` in the Python package `scipy` performed our t-tests.

Table S3. ConsAlign's two-sided, paired t-tests to conventional AF tools using the dataset "RNAStralign processed."

Conventional tool	SPS-based p-value	SCI-based p-value
RAF	5.0×10^{-72} (***)	8.0×10^{-7} (***)
LocARNA	1.5×10^{-127} (***)	6.8×10^{-24} (***)
DAFS	3.0×10^{-213} (***)	3.0×10^{-88} (***)
SPARSE	6.6×10^{-206} (***)	1.4×10^{-73} (***)
LinearTurboFold	3.7×10^{-198} (***)	3.9×10^{-250} (***)

Table S4. ConsAlign's two-sided, paired t-tests to conventional AF tools using BRAliBase's dataset 1.

Conventional tool	SPS-based p-value	SCI-based p-value
RAF	1.2×10^{-8} (***)	1.9×10^{-14} (***)
LocARNA	2.1×10^{-10} (***)	1.3×10^{-7} (***)
DAFS	4.1×10^{-8} (***)	5.2×10^{-10} (***)
SPARSE	5.8×10^{-32} (***)	0.006
LinearTurboFold	0.82	6.3×10^{-34} (***)

References

Do,C.B. *et al*. (2005) ProbCons: Probabilistic consistency-based multiple sequence alignment. *Genome Res.*, **15**, 330–340.

Do,C.B., Woods,D.A. and Batzoglou,S. (2006a) CONTRAfold: RNA secondary structure prediction without physics-based models. *Bioinformatics*, **22**, e90–e98.

Do,C.B., Gross,S.S. and Batzoglou,S. (2006b) CONTRAlign: Discriminative Training for Protein Sequence Alignment. In: *Proceedings of the Tenth Annual International Conference on Computational Molecular Biology (RECOMB 2006)*, Italy, pp. 160–174.

Do,C.B., Foo,C. and Batzoglou,S. (2008) A max-margin model for efficient simultaneous alignment and folding of RNA sequences. *Bioinformatics*, **24**, i68–i76.

Li,S. *et al*. (2021) LinearTurboFold: Linear-time global prediction of conserved structures for RNA homologs with applications to SARS-CoV-2. *Proc. Natl. Acad. Sci. U. S. A.*, **118**.

Sato,K. *et al*. (2012) DAFS: simultaneous aligning and folding of RNA sequences via dual decomposition. *Bioinformatics*, **28**, 3218–3224.

Tagashira,M. and Asai,K. (2022) ConsAlifold: considering RNA structural alignments improves prediction accuracy of RNA consensus secondary structures. *Bioinformatics*, **38**, 710–719.

Tan,Z. *et al*. (2017) TurboFold II: RNA structural alignment and secondary structure prediction informed by multiple homologs. *Nucleic Acids Res.*, **45**, 11570–11581.

Will,S., Reiche,K., Hofacker,I.L., Stadler,P.F. and Backofen,R. (2007) Inferring noncoding RNA families and classes by means of genome-scale structure-based clustering. *PLoS Comput. Biol.*, **3**, 680–691.

Will,S. *et al*. (2015) SPARSE: quadratic time simultaneous alignment and folding of RNAs without sequence-based heuristics. *Bioinformatics*, **31**, 2489–2496.

Zhang,H., Zhang,L., Mathews,D.H. and Huang,L. (2020) LinearPartition: Linear-time approximation of RNA folding partition function and base-pairing probabilities. *Bioinformatics*, **36**, i258–i267.
